# Ascites circRNA ASCOR Drives Platinum Resistance of High‐Grade Serous Ovarian Cancer by Facilitating RPA1 Nuclear Translocation

**DOI:** 10.1002/advs.202518922

**Published:** 2026-02-12

**Authors:** Hanyuan Liu, Chenchen Zhu, Xuelin Yao, Yi Liu, Jing Zhu, Yuebo Li, Hanjie Xu, Cheng Peng, Ge Shan, Liang Chen, Ying Zhou

**Affiliations:** ^1^ Department of Obstetrics and Gynecology Core Facility Center The First Affiliated Hospital of USTC Division of Life Sciences and Medicine University of Science and Technology of China Hefei Anhui China; ^2^ Department of Endocrinology Laboratory of Diabetes The First Affiliated Hospital of USTC Division of Life Sciences and Medicine University of Science and Technology of China Hefei China; ^3^ Department of Obstetrics and Gynecology The First Affiliated Hospital of USTC The RNA Institute School of Basic Medical Sciences Center For Advanced Interdisciplinary Science and Biomedicine of IHM Division of Life Science and Medicine University of Science and Technology of China Hefei China; ^4^ Department of Cardiology The First Affiliated Hospital of USTC Anhui Institute of Cardiovascular Diseases The RNA Institute Division of Life Sciences and Medicine University of Science and Technology of China Hefei China

**Keywords:** ascites, circRNA, nuclear translocation, ovarian cancer, platinum resistance

## Abstract

High‐grade serous ovarian carcinoma (HGSOC) remains the most lethal gynecologic malignancy, with platinum resistance posing the major therapeutic barrier. Malignant ascites, a hallmark of advanced HGSOC, correlates with chemoresistance and poor prognosis, yet the contribution of ascites circRNAs in this process remains obscure. Here, we identify ASCOR, a circRNA upregulated in ascites small extracellular vesicles (sEVs) from platinum‐resistant HGSOC patients, predicts poor survival. ASCOR promotes platinum resistance in vitro and in vivo by enhancing cell viability, reducing apoptosis, and alleviating DNA damage, with effects also transferred via sEVs. Mechanistically, ASCOR binds RPA1 and facilitates its NUP153‐dependent nuclear translocation, which recruits the RNA helicase DDX18, suppressing R‐loop formation. Meanwhile, the ASCOR‐RPA1‐DDX18 axis activates PI3K/Akt signaling to promote platinum resistance. Targeting ASCOR with antisense oligonucleotides reverses chemoresistance in mouse models. Our study unveils an ascites circRNA‐driven pathway underlying platinum resistance in HGSOC, suggesting targeting ASCOR is a promising strategy for HGSOC treatment.

## Graphical abstract

High‐grade serous ovarian carcinoma (HGSOC) is plagued by platinum resistance, with malignant ascites closely linked to poor outcomes. Here, we identify ASCOR, a circRNA enriched in ascites small extracellular vesicles (sEVs) from platinum‐resistant HGSOC patients, as a predictor of poor survival. ASCOR enhances platinum resistance in vitro and in vivo by binding RPA1, facilitating its NUP153‐dependent nuclear translocation, recruiting DDX18 to suppress R‐loop formation, and activating the PI3K/Akt pathway. Antisense oligonucleotide‐mediated ASCOR targeting reverses chemoresistance in mouse models, highlighting ASCOR as a promising therapeutic target for overcoming platinum resistance in HGSOC.

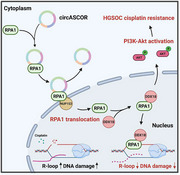

## 1 Introduction

Ovarian cancer (OC) represents one of the most lethal gynecologic malignancies, with 324,398 new cases and 206,839 deaths globally recorded in 2022, ranking sixth among cancer‐related mortalities in women [1, 2]. High‐grade serous carcinoma (HGSOC) accounting for 70–80% of OC cases, is characterized by aggressive progression, frequent recurrence and poor prognosis, often accompanied by malignant ascites—a hallmark of advanced disease [3]. Despite aggressive cytoreductive surgery and platinum‐based chemotherapy, ∼25% of patients recur within 6 months of treatment completion, and over half suffer relapse and develop chemoresistance within 3 years, resulting in advanced HGSOC with a dismal 5‐year survival rate of 30–40% [4]. Malignant ascites, present in >1/3 of patients at diagnosis and nearly all recurrent cases, strongly correlates with poor survival, widespread metastasis, and platinum resistance. Acting as a dynamic tumor microenvironment (TME) reservoir, ascites in HGSOC harbors intrinsically resistant clones and therapy‐adapted cells [5, 6, 7, 8, 9, 10], while concurrently, acellular components in ascites sustain activation of key pro‐resistance pathways [11, 12, 13, 14, 15]. Notably, ascites enables noninvasive longitudinal liquid biopsy sampling, facilitating real‐time monitoring of resistance dynamics [15, 16, 17]. Investigation of platinum resistance mechanisms in ovarian cancer, leveraging ascites as a clinically accessible medium, may thus provide unique insights for identifying novel therapeutic targets.

Platinum resistance in HGSOC, categorized as either intrinsic resistance or acquired resistance [18, 19], is mediated by multifaceted mechanisms, including enhanced DNA damage repair (DDR), dysregulated TME, reduced drug accumulation and evasion of apoptosis [20, 21, 22, 23]. Platinum agents induce cytotoxic DNA cross‐links and double‐strand breaks (DSBs), primarily resolved via homologous recombination repair (HRR), a pathway reliant on replication protein A (RPA). RPA, consisted of RPA1, RPA2 and RPA3 subunits, is a heterotrimeric protein complex found in eukaryotes, essential for DNA replication, repair, recombination and activation of DNA damage checkpoints [24, 25]. Sufficient level of free RPA is critical for the maintenance of genomic integrity, while dysregulated RPA expression or phosphorylation enhances HRR proficiency, enabling tumor cells to survive platinum‐induced genomic stress [26, 27]. RPA1 is the largest and most important subunit of the RPA complex, and is crucial in the nucleus for the cellular response to DNA damage and repair through recruitment and/or activation of proteins involved in the processes, such as RAD51 and ATR [28]. While primarily nuclear, RPA1 can also be found in the cytoplasm, where it helps prevent ssDNA leakage and subsequent activation of cytoplasmic pattern recognition receptors. Dysfunction of RPA1 contributes to genome instability, and further leads to metabolic diseases and cancers [29, 30, 31]. However, the mechanism through which cancer progression is regulated by RPA1 and the pathological functions of RPA1 in the context of platinum resistance still remain unclear.

Circular RNAs (circRNAs) are covalently closed noncoding RNA molecules lacking 5’‐3’ ends and polyadenylated tails [32, 33] with cell type‐ and tissue‐specificity. Most circRNAs are generated in the nucleus and are exported to the cytoplasm in a Exportin 4‐dependent manner [34]. Cytoplasmic circRNAs mainly function through miRNAs sponging, modulation of RNA binding proteins (RBPs), or acting as templates for polypeptides [33, 35]. The physiopathological roles of circRNAs have been extensively studied, and circRNAs are known to play pivotal roles in cancer progression [33, 36, 37, 38]. For example, p53‐induced circFRMD4A enhances cancer cell sensitivity to elesclomol‐induced cuproptosis via metabolic reprogramming [39]; circAURKA promotes tumor proliferation and metastasis in colorectal cancer by inhibiting CTNNB1 degradation via ACLY interaction. In OC, circMUC16 promotes autophagy, invasion, and metastasis by sponging miR‐199a‐5p and binding ATG13 [40]; circPUM1 acts as miRNA sponges to promote tumorigenesis and progression [41]. For the platinum resistance in OC, circNUP50 promotes platinum resistance in ovarian cancer by accelerating p53 ubiquitination via binding p53/UBE2T and sponging miR‐197‐3p to upregulate G3BP1 [42], while circITGB6 stabilizes FGF9 mRNA by scaffolding IGF2BP2‐FGF9 complexes [37]. Of note, malignant ascites is enriched with circRNAs, some of which such as hsa_circ_0000497 and hsa_circ_0000918 contribute to OC metastasis [43]. However, not much is known about the roles of ascites circRNAs in mediating platinum resistance in OC, and the underlying mechanisms on genomic stability maintenance in TME remain poorly defined.

In this study, we have identified Ascites Small extracellular vesicles (sEVs) CircRNA related to Ovarian cancer platinum Resistance (ASCOR) (hsa_circ_0000842), upregulated in platinum‐resistant HGSOC patients, is associated with poor prognosis. ASCOR promotes platinum resistance by increasing cell viability and decreasing apoptosis both in vitro and in vivo. Our mechanistic investigation uncovers that ASCOR facilitates RPA1 nuclear translocation to recruit DDX18 in the nucleus, which impacts genomic stability through suppressing R‐loop formation and thus alleviating DNA damage. Moreover, we have also deciphered, with a series of bioinformatics and cellular analysis, ASCOR‐RPA1‐DDX18 activates PI3K/Akt pathway to promote platinum resistance in HGSOC. Our study unveils the significance of ascites sEV circRNAs ASCOR in mediating chemoresistance in HGSOC, and provides insight into both circRNA‐mediated regulatory networks and the potential applications of circRNA targeted therapy.

## 2 Results

### 2.1 ASCOR is Upregulated in Platinum‐Resistant HGSOC Patients’ Ascites and Correlates with Poor Prognosis

**FIGURE 1:**
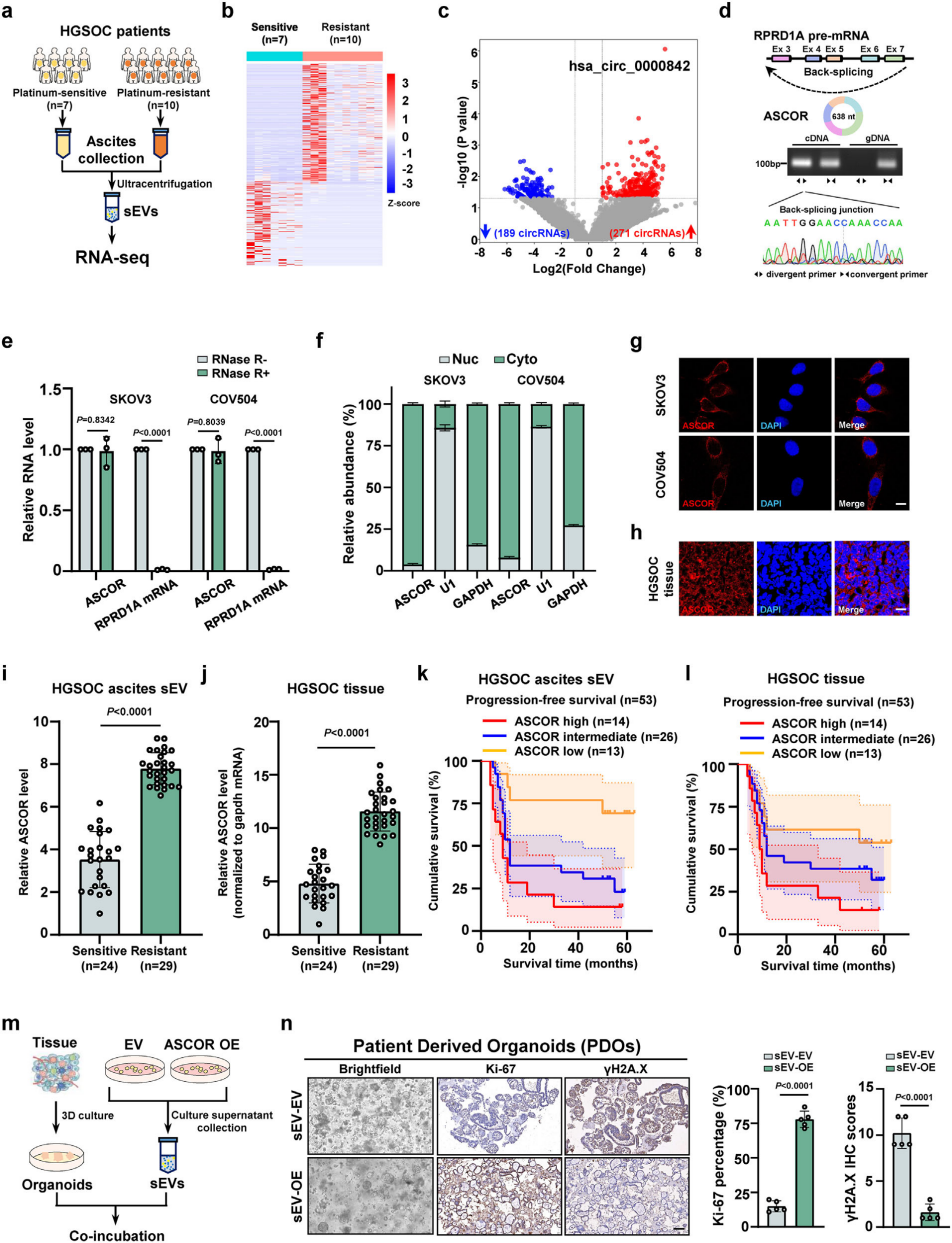
ASCOR is upregulated in platinum‐resistant high‐grade serous ovarian carcinoma (HGSOC) patients’ ascites and correlates with poor prognosis. a) Workflow of isolating small extracellular vesicles (sEVs) from platinum‐sensitive and ‐resistant HGSOC patients ascites for RNA‐seq. b, c) Heatmap (b) and Volcano plot (c) of the differentially expressed circRNAs in RNA‐seq data of ascites sEVs from platinum‐sensitive and ‐resistant HGSOC patients. d) Sanger sequencing of back‐splicing junction site for ASCOR. e) Relative RNA levels of ASCOR and RPRD1A mRNA with or without RNase R digestion in SKOV3 and COV504 cells. f) Subcellular localization of ASCOR in SKOV3 and COV504 cells was assessed by RT‐qPCR analysis after nucleocytoplasmic distribution, GAPDH (a cytoplasmic RNA) and U1 (a nuclear RNA) were included as fractionation controls. g, h) Representative FISH images of ASCOR in SKOV3, COV504 cells (g) and HGSOC patient tissues (h). DAPI, blue; ASCOR, red, scale bar = 10 µm. i, j) Levels of ASCOR in HGSOC ascites sEVs (i) and tissues (j) from cisplatin‐sensitive HGSOC patients (*n* = 24) and cisplatin‐resistant HGSOC patients (*n* = 29). k, l) Progression‐free survival (PFS) analysis of the HGSOC patients was performed in the ASCOR‐high/intermediate/low ascites sEVs (k) and tissues (l) group. For ascites sEVs group: ASCOR high vs. ASCOR intermediate, *p*  =  0.1370; ASCOR high vs. ASCOR low, *p*  =  0.0013; ASCOR intermediate vs. ASCOR low, *p*  =  0.0135. For tissues group: ASCOR high vs. ASCOR intermediate, *p*  =  0.1115 ASCOR high vs. ASCOR low, *p*  =  0.0261; ASCOR intermediate vs. ASCOR low, *p*  =  0.2850. m) Workflow of generating HGSOC patient derived organoids (PDOs). n) Representative images of brightfield, Ki‐67 and γH2A.X immunohistochemical (IHC) staining of HGSOC PDOs, scale bar = 50 nm. The quantification of Ki‐67 positive percentage and γH2A.X IHC scores in each group. ASCOR high: high ASCOR level; ASCOR low: low ASCOR level; OE: overexpression; EV: empty control; sEV‐OE: sEVs‐mediated ASCOR overexpression; sEV‐EV: sEVs‐mediated corresponding empty control. For **e**–**l** and **n**, data are from three independent experiments and shown as mean ± SD. *p* values are from unpaired two‐sided Student's *t*‐test. *p* < 0.05 was considered statistically significant.

In order to investigate the functional roles of ascites circRNAs in mediating platinum response in HGSOC, we first set out to isolate and purify the ascites‐derived sEVs from the ascites of HGSOC patients, considering that sEVs are key contributors to ascites RNA pools. Ascites sEVs from patients with platinum‐sensitive (*n* = 7) and platinum‐resistant (*n* = 10) HGSOC were collected and characterized using TEM, western blotting, and NTA, respectively (Figure 1a and Figure S1a–c). We then performed ribosomal RNA (rRNA)‐depleted RNA sequencing of those ascites sEVs. A total of 460 dysregulated circRNAs were identified in ascites sEVs, of which 271 circRNAs were significantly upregulated and 189 were downregulated (|log2(fold change) | > 1.0, *p* < 0.05) in sEVs from platinum‐resistant patients as compared to those in platinum‐sensitive group. ASCOR, derived from the exons 3–7 of *RPRD1A* gene with a length of 638 nt, emerged as the most significantly upregulated circRNA candidate (with average fold change of 5.8, *p* < 0.0001) (Figure 1b,c). The back‐splicing junction (BSJ) site of ASCOR was amplified using divergent primers and confirmed by Sanger sequencing in SKOV3 cells, an ovarian ascites‐origin OC cell line (Figure 1d). No product was amplified from genomic DNA using divergent primers (Figure 1d). Resistance to RNase R digestion confirmed the closed‐loop structure of ASCOR in both SKOV3 and COV504 cell lines, two cell lines commonly used in OC sEV research [44, 45, 46] (Figure 1e). The subcellular localization of ASCOR was examined using nucleocytoplasmic fractionation followed by RT‐qPCR (Figure 1f) and RNA fluorescence in situ hybridization (FISH) which demonstrated that ASCOR predominantly localized in the cytoplasm in both cell lines, as well as in primary HGSOC tumor tissues (Figure 1g,h).

To address whether ASCOR exerts effects primarily through autocrine or paracrine pathways, we detected ASCOR levels to include HGSOC ascites sEV, and matched primary tumor tissues, omentum, peritoneum tissues. Results showed that ASCOR was significantly upregulated in ascites sEV and primary tumor tissues, while remaining at low levels in omentum and peritoneum (Figure S1d). Furthermore, we overexpressed ASCOR in nontumor HEK293T cells, and results showed that while ASCOR was markedly elevated in HEK293T cells themselves, its level in culture supernatant‐derived sEVs only slightly increased (Figure S1e, f), demonstrating that ASCOR is predominantly derived from autocrine secretion by HGSOC cells, and only tumor‐related tissues exhibit high ASCOR levels. RNA binding protein HNRNPA2B1 is reported to facilitate sEVs cargo packaging [47, 48], and we also found via RNA immunoprecipitation (RIP) assays that HNRNPA2B1 interacted with ASCOR (Figure S1g), and knockdown of HNRNPA2B1 significantly reduced ASCOR levels in sEVs from SKOV3 and COV504 cell culture supernatants (Figure S1h, i), suggesting that HNRNPA2B1 is at least one key mediator of ASCOR packaging into sEVs.

We next analyzed the clinicopathological features of ASCOR, and found that ASCOR levels were significantly upregulated in both ascites‐derived sEVs and matched HGSOC tissues from platinum‐resistant HGSOC patients (*n* = 29) compared to those from platinum‐sensitive patients (*n* = 24) (Figure 1i,j). Then these 53 HGSOC patients were divided into three groups according to ASCOR levels: higher ASCOR levels (ASCOR high, *n* = 14), intermediate ASCOR levels (ASCOR intermediate, *n* = 26) and lower ASCOR levels (ASCOR low, *n* = 13). Results demonstrated that higher ASCOR levels in both ascites‐derived sEVs and their matched HGSOC tissues predicted significantly shorter progression‐free survival (PFS), compared to those with lower ASCOR levels (Figure 1k,l). To further confirm the impact of ASCOR on HGSOC chemoresistance, SKOV3 and COV504 stable cell lines with ASCOR overexpression were established, and RPRD1A mRNA levels were not affected upon ASCOR overexpression (Figure S1j). Next, tumor tissues procured during primary cytoreductive surgery were then utilized to establish patient‐derived organoids (PDOs) models via in vitro 3D culture. These PDOs were coincubated with sEVs isolated from the supernatant of ASCOR OE and the empty control SKOV3 cell lines, respectively (Figure 1m). Immunohistochemical (IHC) analysis of treated PDOs revealed that overexpression of ASCOR significantly increased the levels of Ki‐67 (a proliferation marker) and decreased the levels of γH2AX (a DNA damage marker) (Figure 1n).

Collectively, these results demonstrated that ASCOR was upregulated in both ascites‐derived sEVs and their matched tumor tissues from HGSOC platinum‐resistant specimens, and high level of ASCOR was associated with poor prognostic outcome.

### 2.2 ASCOR Promotes Platinum Resistance In Vitro

**FIGURE 2:**
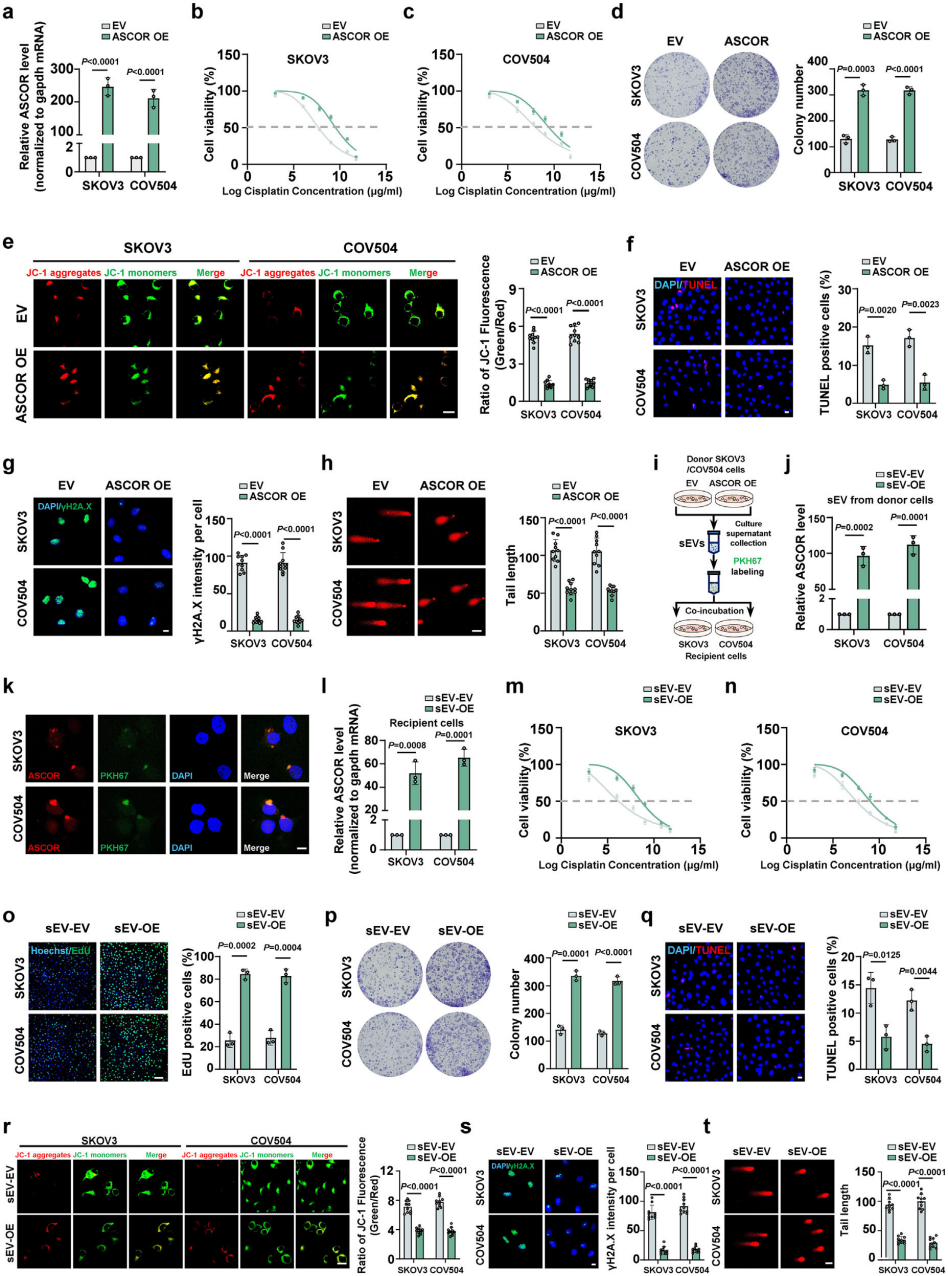
ASCOR promotes platinum resistance in vitro. a) RT‐qPCR validation for the ASCOR overexpression in SKOV3 and COV504 cells. b, c) Cell viability assessed by CCK8 assay upon ASCOR overexpression in SKOV3 (b) and COV504 (c) cells with cisplatin treatment at indicated concentrations for 36 h. IC50: SKOV3 control group, 6.025 µg/mL; SKOV3 ASCOR overexpression group, 9.194 µg/mL; COV504 control group, 6.693 µg/mL; SKOV3 ASCOR overexpression group, 9.096 µg/mL. d) Colony formation assay upon ASCOR‐overexpression in SKOV3 and COV504 cells. e, f) JC‐1 staining (e) and TUNEL staining (f) upon ASCOR overexpression in SKOV3 and COV504 cells, scale bar = 20 µm. g) immunofluorescence (IF) staining of γH2A.X upon ASCOR‐overexpression in SKOV3 and COV504 cells, scale bar = 10 µm. h) Comet assay upon ASCOR‐overexpression in SKOV3 and COV504 cells, scale bar = 50 µm. i) Workflow of small extracellular vesicles (sEVs) mediated transfer of ASCOR from donor SKOV3 and COV504 cells to recipient wild‐type SKOV3 and COV504 cells. j) RT‐qPCR validation for ASCOR overexpression in sEVs of donor cells. k) Colocalizing visualization by ASCOR FISH and IF staining of PKH67 in recipient SKOV3 and COV504 cells. l) RT‐qPCR validation for ASCOR overexpression in recipient cells after 24 h coincubation. DAPI, blue; ASCOR, red; PKH67, green; scale bar = 10 µm. m, n) Cell viability by CCK8 assay in recipient SKOV3 (m) and COV504 cells (n) with cisplatin treatment at indicated concentrations for 36 h. IC50: SKOV3 sEV‐EV group, 4.519 µg/mL; SKOV3 sEV‐OE group, 7.450 µg/mL; COV504 sEV‐EV group, 5.833 µg/mL; COV504 sEV‐OE group, 8.215 µg/mL. o, p) Validation of cell proliferation by EdU assays (o) and colony formation assays (p) in recipient SKOV3 and COV504 cells, scale bar = 100 µm. q, r) TUNEL staining (q) and JC‐1 (r) staining for mitochondrial membrane potential measurement in recipient SKOV3 and COV504 cells. scale bar = 20 µm. s) IF staining of γH2A.X in recipient SKOV3 and COV504 cells, scale bar = 10 µm. t) Comet assay in recipient SKOV3 and COV504 cells, scale bar = 50 µm. OE: overexpression; EV: corresponding empty control; sEV‐OE: sEVs‐mediated ASCOR overexpression; sEV‐EV: sEV‐mediated corresponding empty control. For d–h and o–t, samples were treated with cisplatin at 6 µg/mL. For a–h and j–t, data are from three independent experiments and shown as mean ± SD. *p* values are from unpaired two‐sided Student's *t*‐test. *p* < 0.05 was considered statistically significant.

We then assessed the functional roles of ASCOR in promoting chemoresistance in HGSOC. Upon platinum treatment, ASCOR overexpression significantly enhanced cell viability (Figure 2a–c), and colony formation capacity (Figure 2d) in both stable cell lines. Mitochondrial membrane potential (MMP) analysis using JC‐1 staining further revealed that ASCOR overexpression led to a significant decrease in the green/red fluorescence intensity ratio compared to the control group, indicating preserved MMP and reduced mitochondrial depolarization—a hallmark of early apoptotic events (Figure 2e). TUNEL assays demonstrated a significant decrease in apoptotic cells upon ASCOR overexpression in both cell lines (Figure 2f). ASCOR overexpression also contributes to reduced DNA damage as evidenced by reduced γH2AX intensity in immunofluorescence (IF) and decreased tail length in comet assays (Figure 2g,h). Western blot analysis showed significantly reduced levels of γH2AX in the ASCOR overexpression group, compared to the control group (Figure S2a).

Considering ASCOR was first identified in ascites sEVs from HGSOC patients, we then investigated whether ASCOR pro‐chemoresistance effects could be transferred through sEVs. sEVs were isolated from the culture media of ASCOR overexpression and the control cells respectively (Figure 2i), and ASCOR overexpression in sEVs was validated (Figure 2j). sEVs were then labeled with PKH67, a fluorescent dye commonly used for the labeling and visualization of sEVs, and were then subjected to coincubation with SKOV3 and COV504 wild‐type (WT) recipient cells for 24 h. Colocalization signals between IF tracking of PKH67 and ASCOR FISH within recipient cells revealed successful sEV internalization and the presence of transferred ASCOR (Figure 2k). To assess the efficiency of sEV‐transfer of ASCOR, RT‐qPCR was used to validate the overexpression of ASCOR, which showed significantly elevated ASCOR levels in both recipient cells (Figure 2l). We further examined the functional effects of sEV‐transferred ASCOR in the recipient cells. Compared to the control groups, cells with sEV‐mediated ASCOR overexpression exhibited significantly enhanced cell viability upon cisplatin treatment at different doses (Figure 2m,n). This pro‐survival effect was corroborated by increased cell proliferation capability, as demonstrated by EdU incorporation and colony formation assays (Figure 2o,p). Similarly, TUNEL assays and MMP assay showed sEV‐mediated ASCOR overexpression significantly suppressed cell apoptosis (Figure 2q,r). IF of γH2AX and comet assay further showed significant reductions in DNA damage with decreased γH2AX and tail length (Figure 2s,t). Western blot analysis also showed significantly reduced levels of γH2AX in the sEV‐ASCOR overexpression group of both recipient cells, compared to the control group (Figure S2b). Together, these findings suggested that ASCOR promoted platinum resistance in vitro by increasing cell proliferation and reducing apoptosis; and these effects could be transferred by sEV‐mediated ASCOR overexpression.

### 2.3 ASCOR Promotes Platinum Resistance In Vivo

**FIGURE 3:**
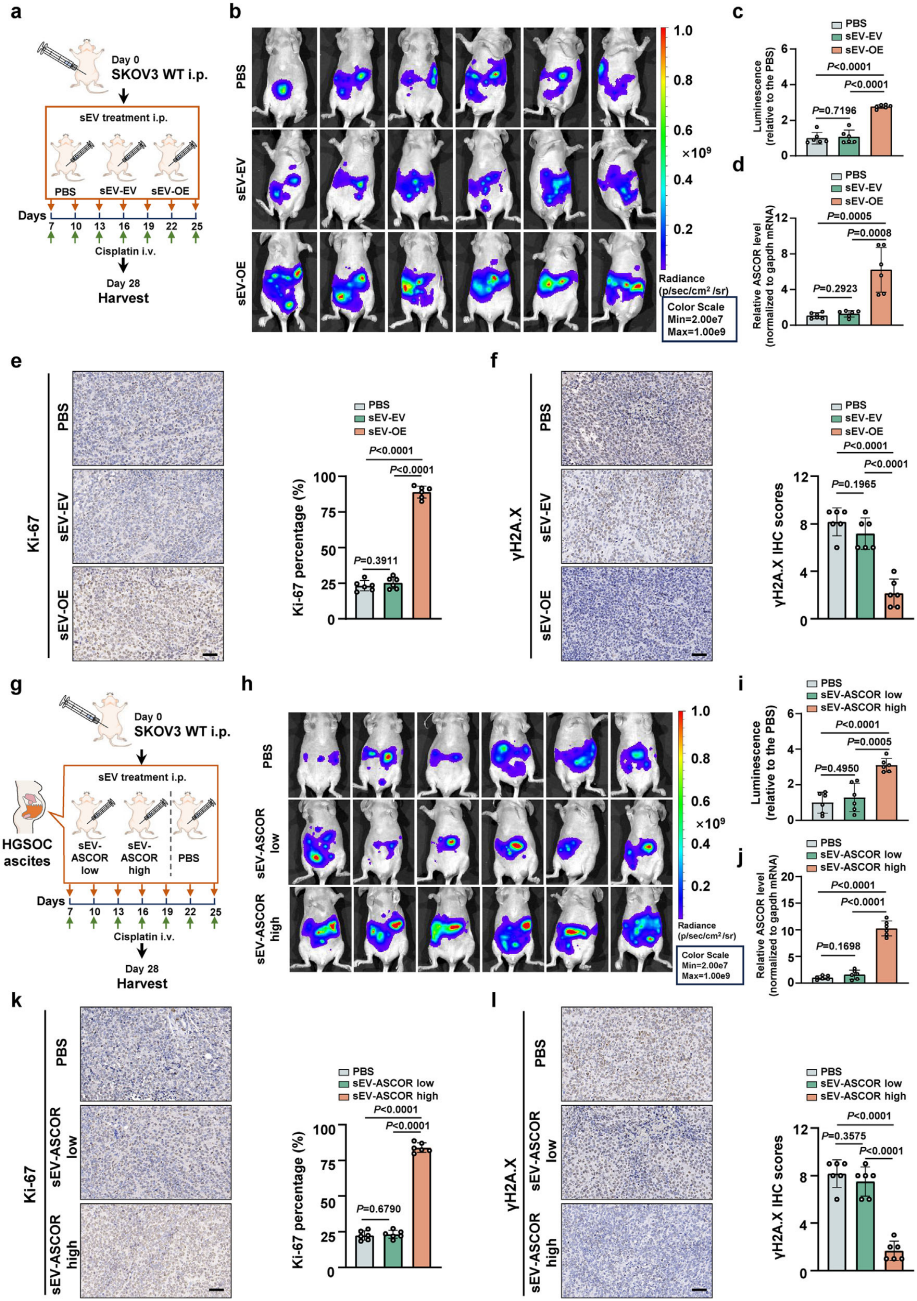
ASCOR promotes platinum resistance in vivo. a) Workflow for the generation of high‐grade serous ovarian carcinoma (HGSOC) xenograft mice model. Mice were treated with 10 µg indicated small extracellular vesicles (sEVs) i.p. b) Representative bioluminescent images of the xenograft nude mice treated with PBS, sEVs‐mediated ASCOR‐overexpression or empty vector control i.v. c) quantification of bioluminescent imaging signal intensities of the mice in each group. *n* = 6 in each group. d) RT‐qPCR validation of relative ASCOR levels in xenograft tumors. e, f) Immunohistochemistry (IHC) analysis of Ki‐67 (e) and γH2A.X (f) of the tumors dissected from each group with quantification shown with the bar figures. Scale bar = 50 µm. g) Workflow for the xenograft nude mice treated with PBS or 10 µg ascites sEVs from HGSOC patients with high or low ASCOR levels. h) Representative bioluminescent images of the xenograft nude mice treated with PBS, sEVs derived from ASCOR‐low or ASCOR‐high HGSOC patients’ ascites i.p. i) quantification of bioluminescent imaging signal intensities. *n* = 6 for each group. j) RT‐qPCR validation for the relative ASCOR levels in xenograft tumor. k, l) IHC analysis of Ki‐67 (k) and γH2A.X (l) in the tumors dissected from each group with quantification shown with the bar figures. Scale bar = 50 µm. For all the mice experiments, mice were treated with 5 mg/kg cisplatin once every 3 days before the mice were sacrificed. sEV‐OE: sEVs‐mediated ASCOR overexpression; sEV‐EV: sEVs‐mediated corresponding empty control; sEV‐ASCOR low: sEVs from HGSOC patient ascites with ASCOR low levels; sEV‐ASCOR high: sEVs from HGSOC patient ascites with ASCOR high levels. For d–f and g–l, data are from three independent experiments and shown as mean ± SD. *p* values are from unpaired two‐sided Student's *t*‐test. *p* < 0.05 was considered statistically significant.

To evaluate the pro‐resistance functions of ASCOR in vivo, SKOV3 WT cells were intraperitoneally implanted into the nude mice models. After 1 week of tumor engraftment, mice received intraperitoneal injections of sEVs isolated from either ASCOR overexpression or the control SKOV3 stable cells, following additional 3 weeks of cisplatin intravenous treatment at the indicated time before the mice were sacrificed (Figure 3a). Bioluminescence imaging revealed that mice with sEV‐ASCOR overexpression exhibited significantly stronger tumor‐derived luciferase signals compared to the EV group and the PBS‐treated blank group (Figure 3b,c). Tumors from all groups were dissected for further analysis. Significantly higher ASCOR levels were validated in tumors from sEV‐ASCOR overexpression group (Figure 3d). IHC staining of the tumors confirmed that ASCOR overexpression group exhibited an increased level of Ki‐67 and decreased level of γH2AX (Figure 3e,f).

To further validate the physiopathological relevance of sEV‐ASCOR, sEVs were isolated from clinical ascites of HGSOC patients from ASCOR‐high and ASCOR‐low groups respectively, and were administered intraperitoneally to the nude mice 1 week after tumor engraftment by SKOV3 WT cells. Cisplatin intravenous treatment was conducted at the indicated time before the mice were sacrificed (Figure 3g). Results showed that nude mice administered sEVs from ASCOR‐high group exhibited stronger tumor bioluminescence signals (Figure 3h,i) and significantly elevated ASCOR levels in tumor xenografts (Figure 3j). IHC staining of tumor xenografts also showed an increased Ki‐67 level and a decreased γH2AX level (Figure 3k,l). These data demonstrated that ASCOR, delivered via sEVs, promotes tumor growth and enhances resistance to cisplatin in vivo in both xenograft models treated with sEVs from ASCOR‐overexpression cells and those from clinical ascites of HGSOC patients with high ASCOR levels.

### 2.4 ASCOR Interacts with RPA1 Protein

**FIGURE 4:**
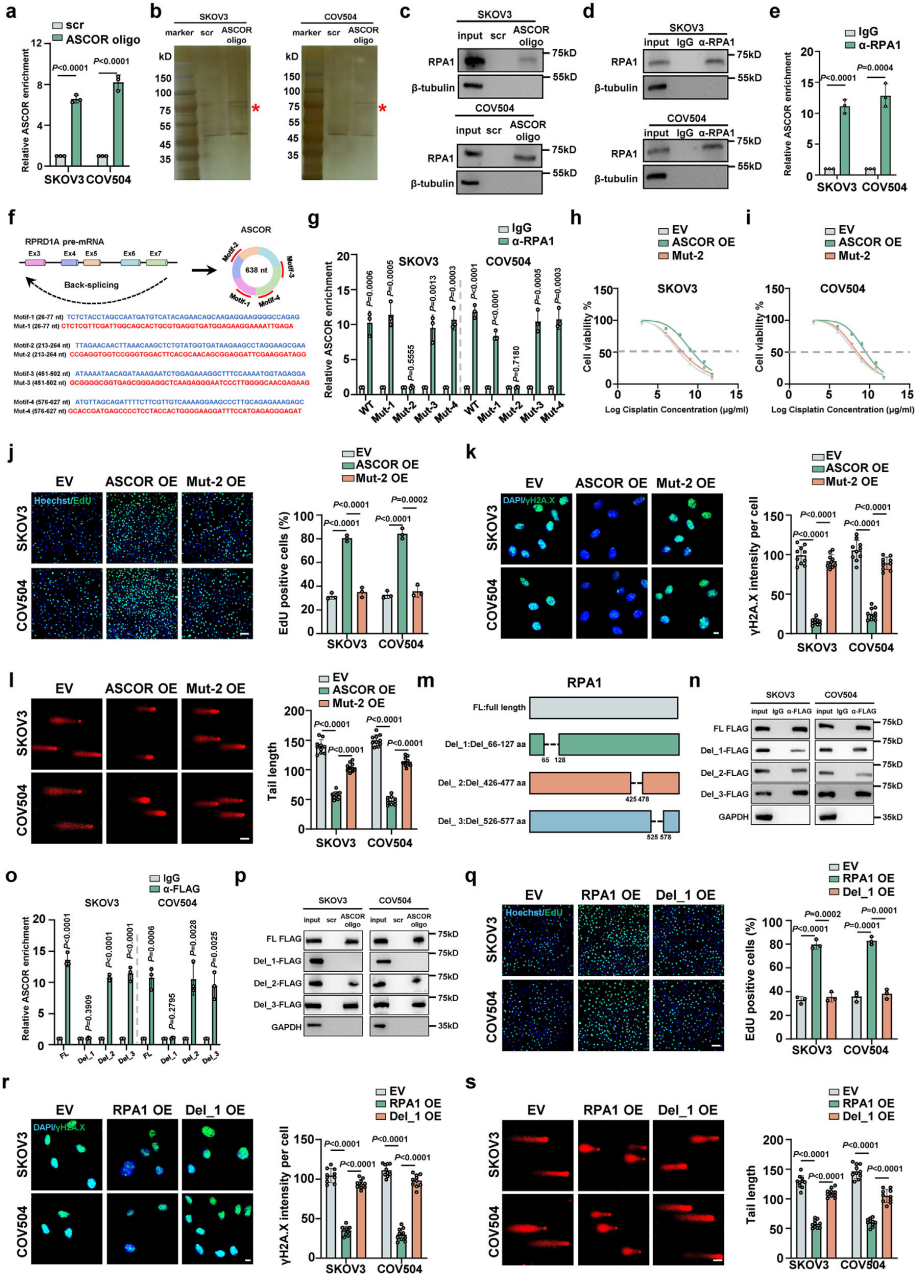
ASCOR interacts with RPA1 protein. a, b) The pulldown efficiency of ASCOR (a) and silver staining of ASCOR pulled‐down proteins (b) in SKOV3 and COV504 cells, “_*_” denotes the RPA1 protein. scr, scramble control oligo; ASCOR oligo, biotin‐labeled ASCOR oligo. c) Western blot validation of RPA1 in ASCOR pulldown in SKOV3 and COV504 cells. d, e) Western blot validation of RPA1 RIP (d) and relative enrichment of ASCOR (e) in SKOV3 and COV504 cells. f) Demonstration of predicted binding motifs of ASCOR to RPA1by catRAPID database. g) Relative enrichment of ASCOR and the mutants in RPA1 RIP in SKOV3 and COV504 cells. h, i) Cell viability by CCK8 assay in SKOV3 (h) and COV504 (i) cells upon ASCOR WT, ASCOR or Mut‐2 overexpression, respectively. IC50: SKOV3 control group, 6.091 µg/mL; SKOV3 ASCOR overexpression group, 8.833 µg/mL; SKOV3 ASCOR Mut‐2 overexpression group, 6.694 µg/mL; COV504 control group, 6.731 µg/mL; COV504 ASCOR overexpression group, 9.162 µg/mL; COV504 ASCOR Mut‐2 overexpression group, 7.240 µg/mL. j) EdU assays in SKOV3 and COV504 cells upon ASCOR WT, ASCOR or Mut‐2 overexpression, respectively, scale bar = 100 µm. k) Immunofluorescence (IF) staining of γH2A.X in SKOV3 and COV504 cells upon ASCOR WT, ASCOR or Mut‐2 overexpression, respectively, scale bar = 10 µm. l) Comet assays in SKOV3 and COV504 cells upon ASCOR WT, ASCOR or Mut‐2 overexpression, respectively, scale bar = 50 µm. m) Demonstration of predicted binding regions of RPA1 to ASCOR by catRAPID database, and FLAG‐tagged truncations with RPA1 indicated deletion were constructed. n, o) Western blot validation of anti‐FLAG RIP (n) and relative enrichment of ASCOR (o) in SKOV3 and COV504 cells. p) Western blot validation of full‐length (FL) RPA1 and the deletions in ASCOR pulldown in SKOV3 and COV504 cells. q) EdU assays upon RPA1 WT or Del_1 overexpression in SKOV3 and COV504 cells, scale bar = 100 µm. r) IF staining of γH2A.X upon RPA1 WT or Del_1 overexpression in SKOV3 and COV504 cells, scale bar = 10 µm. s) Comet assays upon RPA1 WT or Del_1 overexpression in SKOV3 and COV504 cells, scale bar = 50 µm. ASCOR OE: ASCOR overexpression; Mut2 OE: ASCOR‐Mut 2 overexpression; RPA1 OE: RPA1 overexpression; D1 OE: Del_1 overexpression; EV: corresponding empty control. For j–l and q–s, samples were treated with cisplatin at 6 µg/mL. For a–e, g—l, and n–s, data are from three independent experiments and shown as mean ± SD. *p* values are from unpaired two‐sided Student's *t*‐test. *p* < 0.05 was considered statistically significant.

We set out to elucidate the functional mechanisms of ASCOR through exploring the potential interacting molecules. RNA immunoprecipitation (RIP) of AGO2, mediator of interaction between target RNA and miRNA, showed no enrichment of ASCOR (Figure S3a). ASCOR also displayed low coding possibilities (*R* < 1.6 with no open reading frame) in circRNADb predicting database. Therefore, ASCOR is noncoding and does not function as miRNA sponge. To investigate the potential interacting proteins, we performed RNA pulldown in both cells with biotinylated oligo against the BSJ of ASCOR with high efficiency (Figure 4a). The co‐pulled down protein candidates were separated by SDS‐PAGE and were subjected to silver staining to unveil the specific ASCOR‐binding band followed by mass spectrometry (MS) (Figure 4b). Among the top 3 protein candidates (Figure S3b), RPA1 (replication protein A 70 kDa DNA‐binding subunit), known for its functions in DNA replication, repair, and genomic stability maintenance, was identified as the ASCOR‐interacting protein with high possibility (Figure 4c and Figure S3c). High expression of RPA1 is associated with unfavorable overall survival (OS) and PFS in OC (Figure S3d–f). Subsequent western blot following ASCOR pulldown validated that RPA1 could be co‐pulled down by specific ASCOR probe, and RPA1 RNA immunoprecipitation (RIP) assays in both cells validated that ASCOR was significantly enriched (Figure 4d,e). Given the cytoplasmic localization of ASCOR, cytoplasmic fraction was used to further verify the ASCOR‐RPA1 interaction by cytoplasmic ASCOR pulldown and RPA1 RIP (Figure S3g–j), which further confirmed ASCOR‐RPA1 cytoplasmic interaction.

To map the binding sites of RPA1 in ASCOR, catRAPID, a tool for predicting the protein‐interacting region with RNAs, was used in which four binding motifs of RPA1 in ASCOR were identified with high confidence, and these motifs were thus mutated (Figure 4f). Results via RPA1 RIP showed that mutations in motif‐2 (Mut‐2) in ASCOR decreased the interaction with RPA1 in both cells, indicating that this motif was necessary for ASCOR binding to RPA1 (Figure 4g). Overexpression of Mut‐2 abrogated the pro‐resistant phenotype of wild‐type ASCOR, with compromised cell viability (Figure 4h,i), reduced proliferative capacity (Figure 4j), and increased DNA damage (Figure 4k, l) in both SKOV3 and COV504 cells. In parallel, FLAG‐tagged RPA1 truncation mutants were generated with deletion of three predicted binding domains Del_1 (Del_66‐127 aa), Del_2 (Del_426‐477 aa), and Del_3 (Del_526‐577 aa) via catRAPID (Figure 4m). Anti‐FLAG RIP analysis demonstrated that Del_1 abolished the RPA1 interaction with ASCOR (Figure 4n,o), while ASCOR pulldown also demonstrated that Del_1 failed to be co‐pulled down with ASCOR (Figure 4p) indicating that the 66–127 aa region of RPA1 might be indispensable for ASCOR binding. These results demonstrated that ASCOR exerted its pro‐resistance functions through the interaction with RPA1 at critical regions.

To investigate the functional roles of RPA1 in OC platinum resistance, we established SKOV3 and COV504 stable cell lines with RPA1‐overexpression (Figure S3k, l). Upon cisplatin treatment, RPA1 overexpression significantly led to enhanced cellular viability (Figure S4a, b), colony formation capacity (Figure S4c), and cell proliferation (Figure S4d). RPA1 overexpression also resulted in reduced apoptosis (Figure S4e) and decreased DNA damage (Figure S4f, g), as evidenced by MMP, γH2AX IF and comet assays. In xenograft nude mice treated with cisplatin, intraperitoneal administration of RPA1‐overexpression SKOV3 cells exhibited enhanced tumor growth (Figure S4h–j), with IHC staining of elevated Ki67 and reduced γH2AX (Figure S4k). Overexpression of Del_1 reversed the pro‐resistance phenotype of RPA1 WT, with decreased proliferation capability (Figure S4l, m and Figure 4q), and increased DNA damage (Figure 4r, s) caused in both SKOV3 and COV504 cells upon cisplatin treatment, further confirming RPA1 functions in promoting platinum resistance.

### 2.5 ASCOR Promotes Nuclear Translocation of RPA1 Protein

**FIGURE 5:**
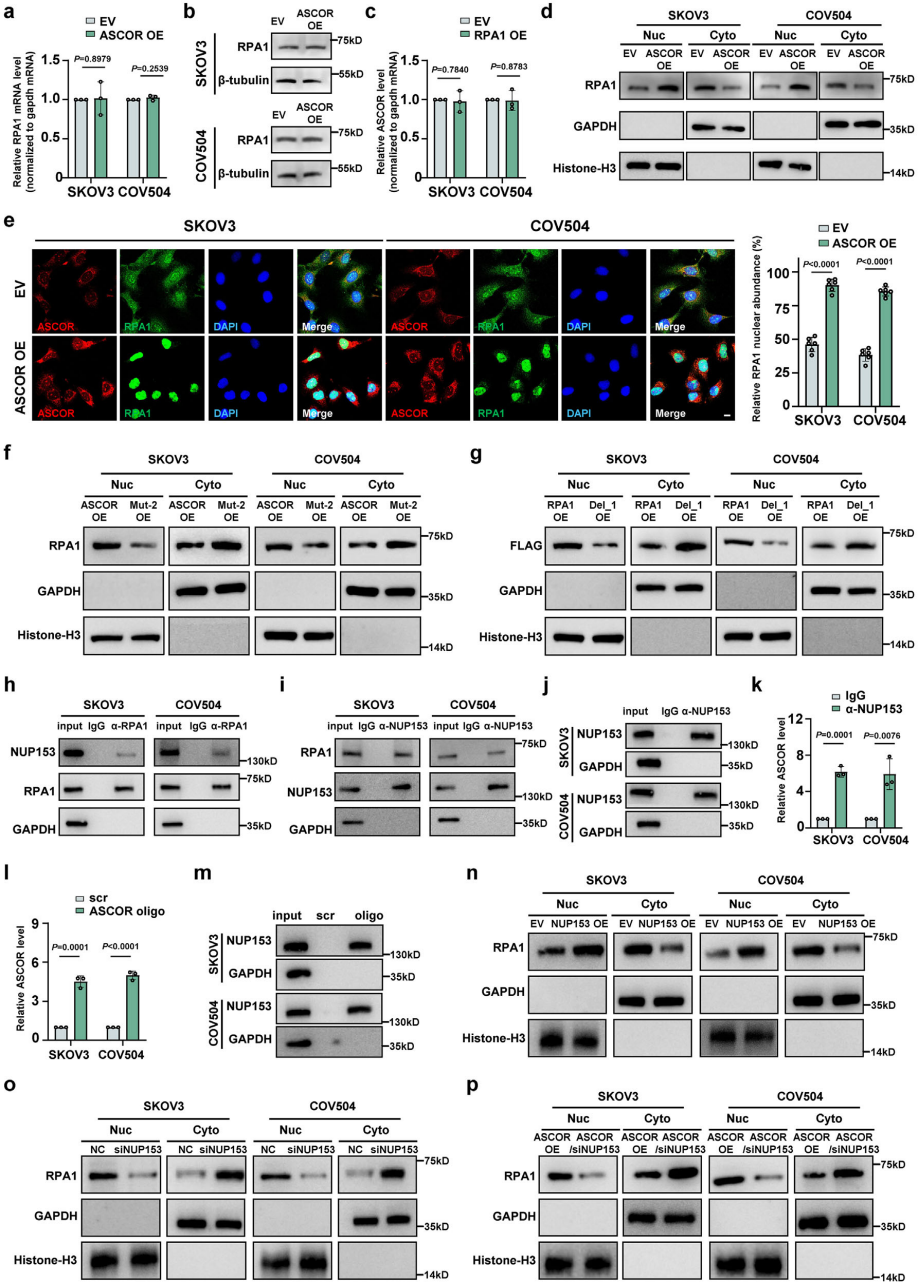
ASCOR promotes nuclear translocation of RPA1 protein. a, b) RPA1 mRNA and protein levels examined by RT‐qPCR (a) and Western blot (b) upon ASCOR‐overexpression in SKOV3 and COV504 cells. c) ASCOR level examined by RT‐qPCR upon RPA1‐overexpression in SKOV3 and COV504 cells. d) Western blot of nuclear and cytoplasmic of RPA1 upon ASCOR‐overexpression in SKOV3 and COV504 cells. GAPDH and Histone‐H3 were used as cytoplasmic and nuclear references, respectively. e) FISH staining of ASCOR and immunofluorescence (IF) staining of RPA1 upon ASCOR‐overexpression in SKOV3 and COV504 cells, scale bar = 10 µm. Relative RPA1 nuclear abundance was shown. f) Western blot of nuclear and cytoplasmic level of RPA1 upon wildtype ASCOR or ASCOR Mut2 overexpression in SKOV3 and COV504 cells. g) Western blot of nuclear and cytoplasmic level of RPA1 upon RPA1 or RPA1 Del_1 overexpression in SKOV3 and COV504 cells. h, i) The interaction between NUP153 and RPA1 was examined by co‐IP using anti‐RPA1 antibody (h) and anti‐NUP153 antibody (i). j, k) Western blot validation of NUP153 RIP (j) and relative enrichment of ASCOR (k) in SKOV3 and COV504 cells. l, m) The pulldown efficiency of ASCOR (l) and Western blot validation of NUP153 (m) in ASCOR pulldown in SKOV3 and COV504 cells. n, o) Western blot of nuclear and cytoplasmic level of RPA1 in NUP153 overexpressed (n) or knockdown (o) SKOV3 and COV504 cells. p) Western blot of nuclear and cytoplasmic level of RPA1 upon NC or NUP153 knockdown in ASCOR overexpressed SKOV3 and COV504 cells. OE: overexpression; EV: corresponding empty control; NC: corresponding negative control. For a–p, data are from three independent experiments and shown as mean ± SD. *p* values are from unpaired two‐sided Student's *t*‐test. *p* < 0.05 was considered statistically significant.

We further examined the reciprocal regulation of ASCOR and RPA1, in which the mRNA and protein levels of RPA1 were not affected in both cells by ASCOR overexpression (Figure 5a,b), while ASCOR levels remained unaffected upon RPA1 overexpression (Figure 5c), suggesting the functions by their interaction were independent of their level changes. Given the critical roles of RPA1 in the nucleus [29, 30, 31], subcellular fractionation followed by RPA1 western blot revealed that ASCOR overexpression significantly increased nuclear RPA1 levels, while decreased its levels in the cytoplasm (Figure 5d), which was further corroborated by IF‐FISH colocalization analysis (Figure 5e). Either overexpression of ASCOR Mut‐2 or the RPA1 Del_1 reversed RPA1 nuclear translocation effects in both cells (Figure 5f,g), indicating that ASCOR interaction with RPA1 facilitated the nuclear translocation of RPA1.

To elucidate the mechanism of RPA1 nuclear translocation facilitated by ASCOR, RPA1 co‐immunoprecipitation (co‐IP) coupled with MS identified were performed, and candidates with nuclear transport functions were screened based on major reported nuclear transport factors [49]. NUP153, known for its functions in selective nucleocytoplasmic transport across the nuclear pore complex [50], was selected for the analysis (Figure S5a, b). co‐IP assay with RPA1 antibody precipitated NUP153 (Figure 5h), while NUP153 also precipitated RPA1 (Figure 5i). NUP153 RIP for validating ASCOR enrichment and ASCOR pull‐down followed by NUP153 western blot showed that ASCOR also bound to NUP153 (Figure 5j–m). To validate NUP153 function in RPA1 nuclear translocation, we overexpressed NUP153 and observed an increased level of nuclear RPA1 and decreased level of RPA1 in the cytoplasm. NUP153 knockdown posed the opposite effects with more RPA1 retained in the cytoplasm (Figure 5n,o). NUP153 silencing upon ASCOR overexpression reversed the ASCOR‐facilitated RPA1 nuclear translocation (Figure 5p). Collectively, these data demonstrated that ASCOR promotes RPA1 nuclear translocation in a NUP153‐dependent manner.

### 2.6 ASCOR Promotes RPA1‐Mediated Recruitment of DDX18

**FIGURE 6:**
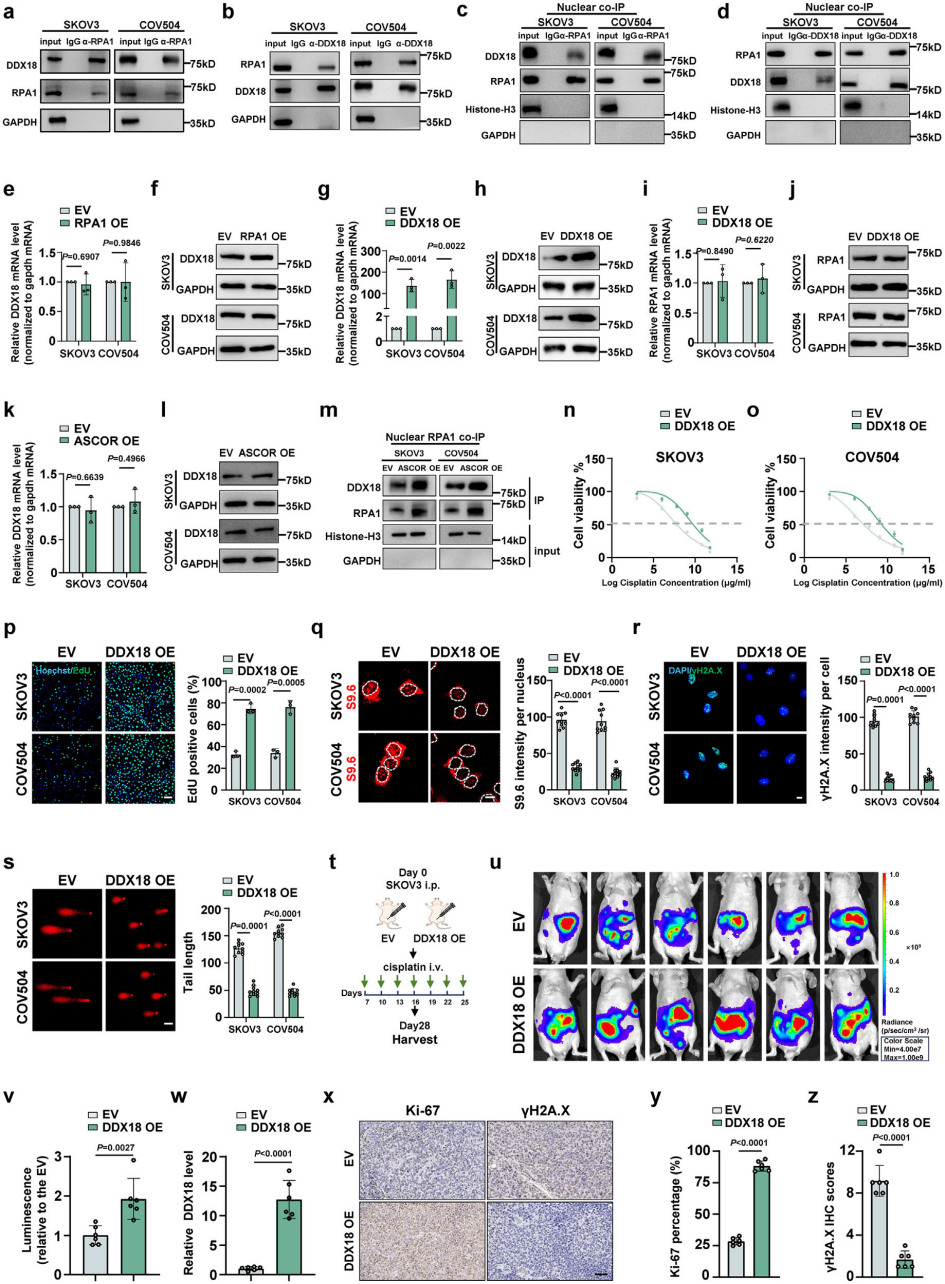
ASCOR promotes RPA1‐mediated recruitment of DDX18. a, b) The interaction between DDX18 and RPA1 was examined by co‐IP and Western blot using anti‐RPA1 antibody (a) and anti‐DDX18 antibody (b). c, d) The interaction between DDX18 and RPA1 was examined by co‐IP using the nuclear fractions of SKOV3 and COV504 cells using anti‐RPA1 antibody (c) and anti‐DDX18 antibody (d). e, f) DDX18 mRNA and protein levels examined by RT‐qPCR (e) and Western blot (f) upon RPA1‐overexpression in SKOV3 and COV504 cells. g, h) Relative DDX18 mRNA and protein levels examined by RT‐qPCR (g) and Western blot (h) upon DDX18‐overexpression inSKOV3 and COV504 cells. i, j) RPA1 mRNA and protein levels examined by RT‐qPCR (i) and Western blot (j) upon DDX18‐overexpression in SKOV3 and COV504 cells. k, l) DDX18 mRNA and protein levels examined by RT‐qPCR (k) and Western blot (l) upon ASCOR‐overexpression in SKOV3 and COV504 cells. m) Western blot for DDX18 in RPA1 co‐IP assay upon ASCOR overexpression. n, o) Cell viability by CCK8 assay upon DDX18 overexpression in SKOV3 (n) and COV504 (o) cells with cisplatin treatment at indicated concentrations for 36 h. IC50: SKOV3 control group, 6.231 µg/mL; SKOV3 DDX18 overexpression group, 9.686 µg/mL; COV504 control group, 5.414 µg/mL; COV504 DDX18 overexpression group, 8.075 µg/mL. p EdU assays upon DDX18‐overexpression in SKOV3 and COV504 cells, scale bar = 100 µm. q) Immunofluorescence (IF) staining of S9.6 upon DDX18‐overexpression in SKOV3 and COV504 cells, scale bar = 10 µm. r) IF staining of γH2A.X upon DDX18‐overexpression in SKOV3 and COV504 cells, scale bar = 10 µm. s) Comet assay upon DDX18‐overexpression in SKOV3 and COV504 cells, scale bar = 50 µm. t) Workflow of HGSOC mice model with DDX18 overexpression. u, v) Representative bioluminescent images of the xenograft intraperitoneal injection with DDX18‐overexpression or control SKOV3 cells (u) and quantification of bioluminescent imaging signal intensities (v), mice were treated with 5 mg/kg cisplatin once every 3 days before the mice were sacrificed. w) Relative DDX18 mRNA levels in xenograft tumor were detected by RT‐qPCR. x) Immunohistochemical (IHC) analysis of Ki‐67 and γH2A.X in the tumors dissected from each group, scale bar = 50 µm. y, z) IHC analysis of Ki‐67 (y) and γH2A.X (z) upon the tumors dissected from each group. The quantification of the markers in each group is shown in the bar figure. Scale bar = 50 µm. OE: overexpression; EV: corresponding empty control. For p–s, samples were treated with cisplatin at 6 µg/mL. For a–s and v–z, data are from three independent experiments and shown as mean ± SD. *p* values are from unpaired two‐sided Student's *t*‐test. *p* < 0.05 was considered statistically significant.

To investigate the functional consequences of ASCOR‐facilitated RPA1 nuclear translocation, RPA1 co‐IP in both cells followed by MS was performed in which three interacting candidates were identified (Figure S6a). DDX18, an RNA helicase that involved in cellular stress responses [51, 52], was coprecipitated by RPA1, while the other two candidates were failed to be detected in RPA1 co‐IP (Figure S6b, c). DDX18, Western blot further showed that DDX18 also coprecipitated RPA1 (Figure 6a, b). Given the nuclear localization of DDX18 and its conserved roles in genome maintenance [51, 53], nuclear fraction was used to further verify the RPA1‐DDX18 interaction by nuclear co‐IP (Figure 6c,d). Neither the mRNA nor protein level of DDX18 was unaffected by RPA1 overexpression (Figure 6e,f), and DDX18 overexpression did not affect mRNA or protein level of RPA1 (Figure 6g–j). Overexpression of ASCOR did not alter the DDX18 mRNA or protein level (Figure 6k,l). Notably, RPA1 co‐IP using nuclear fraction revealed that more DDX18 was recruited to bind with RPA1 upon ASCOR overexpression (Figure 6m), indicating that ASCOR promoting RPA1 nuclear translocation enhances DDX18 recruitment to RPA1 in the nucleus.

Kaplan–Meier analysis revealed that high expression of DDX18 is correlated with poorer OS and PFS in OC (Figure S6d–f). We next examined the functional roles of DDX18 in OC chemoresistance. DDX18 overexpression in both cells significantly enhanced cellular viability (Figure 6n, o) and proliferative capacity (Figure 6p) upon cisplatin treatment. DDX18 is a member of DEAD‐box RNA helicase, which is known to unwind RNA/DNA hybrid promoting R‐loop resolution, and reduces DNA damage caused by excessive R‐loop accumulation preventing genomic instability [53]. To investigate whether enhanced DDX18 recruitment to RPA1 impacts HGSOC chemoresistance by modulating R‐loop accumulation, IF with S9.6, a monoclonal antibody broadly used for detection and study of R‐loop, was performed which showed that DDX18 overexpression decreased the R‐loop formation (Figure 6q). Concurrent decreased DNA damage was also observed as evidenced by reduced γH2AX signal intensity and comet tail length (Figure 6r, s). Intraperitoneal administration of DDX18 overexpression SKOV3 cells in the nude mice upon cisplatin treatment demonstrated that DDX18 overexpression enhanced the platinum resistance, with IHC analysis showed increased Ki‐67 and decreased γH2AX signals (Figure 6t–z). These data confirmed DDX18 functional roles in promoting platinum resistance by enhancing proliferation and genomic stability, and these functions may be enhanced by nuclear recruitment RPA1.

**FIGURE 7:**
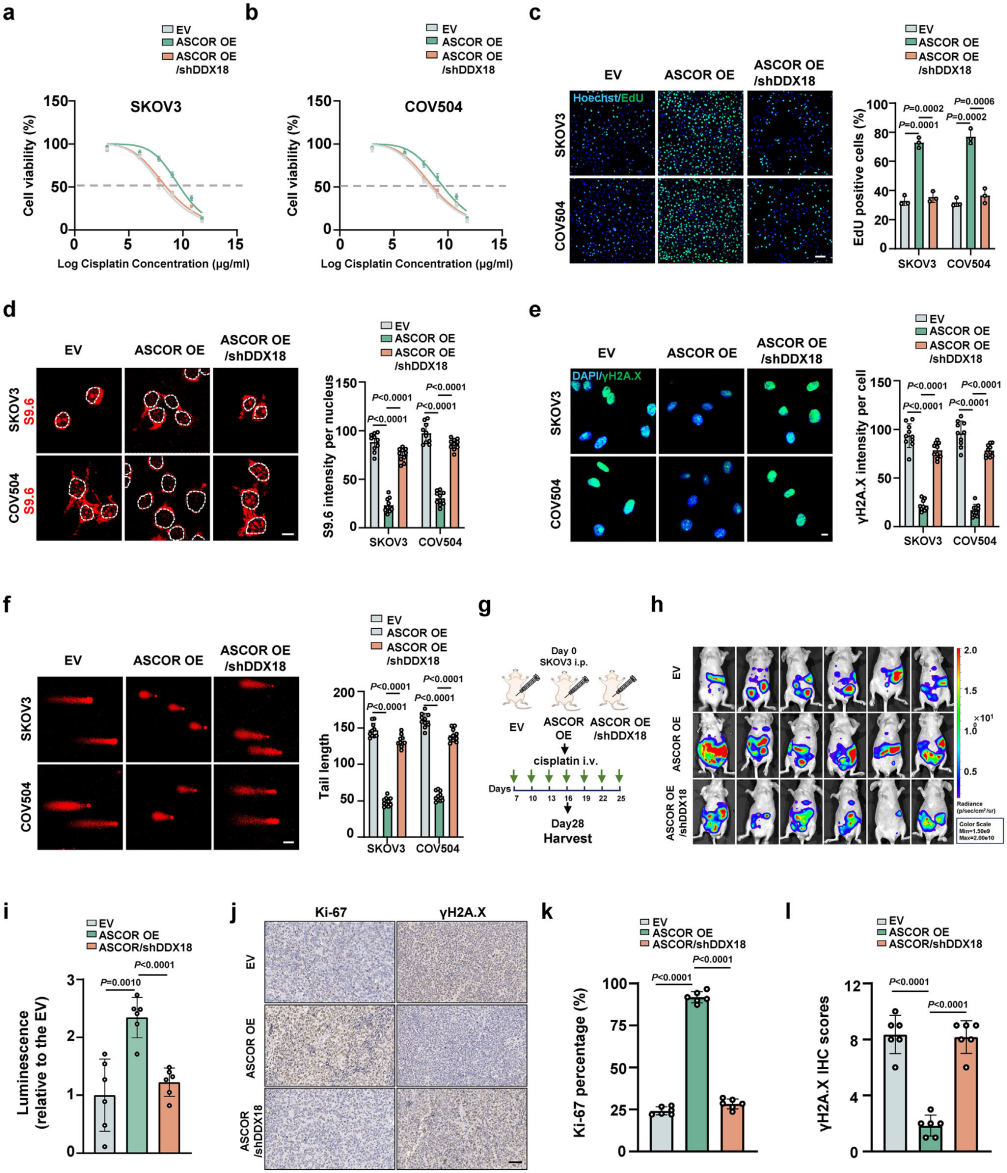
ASCOR‐RPA1‐DDX18 axis promotes platinum resistance. a, b) Cell viability by CCK8 assay upon DDX18 knockdown in SKOV3 (a) and COV504 (b) ASCOR overexpression stable cells with cisplatin treatment at indicated concentrations for 36 h. IC50: SKOV3 control group, 6.797 µg/mL; SKOV3 ASCOR overexpression group, 9.598 µg/mL; SKOV3 DDX18 knockdown upon ASCOR overexpression group, 7.277 µg/mL; COV504 control group, 7.156 µg/mL; COV504 ASCOR overexpression group, 9.332 µg/mL; COV504 DDX18 knockdown upon ASCOR overexpression group, 7.553 µg/mL. c) EdU assays upon DDX18 knockdown in SKOV3 and COV504 ASCOR overexpression stable cells, scale bar = 100 µm. d) Immunofluorescence (IF) staining of S9.6 upon DDX18 knockdown in SKOV3 and COV504 ASCOR overexpression stable cells, scale bar = 10 µm. e) IF staining of γH2A.X upon DDX18 knockdown in SKOV3 and COV504 ASCOR overexpression stable cells, scale bar = 10 µm. f) Comet assays upon DDX18 knockdown in SKOV3 and COV504 ASCOR overexpression stable cells, scale bar = 50 µm. g) Workflow of mice HGSOC model with DDX18 knockdown upon ASCOR overexpression. h, i) Representative bioluminescent images of the xenograft mice model with treatment (h), with quantification of bioluminescent imaging signal intensities (i). Mice were treated with 5 mg/kg cisplatin once every 3 days before the mice were sacrificed. j) Immunohistochemical (IHC) analysis of Ki‐67 and γH2A.X in the tumors dissected from each group, scale bar = 50 µm. k, l) IHC analysis of Ki‐67 (k) and γH2A.X (l) in the tumors dissected from each group. The quantification of the markers in each group is shown with the bar figure. Scale bar = 50 µm. OE: overexpression; EV: corresponding empty control. For c–f, samples were treated with cisplatin at 6 µg/mL. For a–f and j–l, data are from three independent experiments and shown as mean ± SD. *p* values are from unpaired two‐sided Student's *t*‐test. *p* < 0.05 was considered statistically significant.

We next conducted DDX18 knockdown upon ASCOR overexpression in both cells with cisplatin treatment, and observed that DDX18 knockdown abrogated ASCOR pro‐resistance phenotypes with decreased viability (Figure 7a,b), proliferation capacity (Figure 7c), and increased R‐loop formation and DNA damage (Figure 7d–f). Experiments with xenograft mice confirmed that administration of SKOV3 stable cell line with DDX18 knockdown upon ASCOR overexpression attenuated the promoting effects of ASCOR in tumor progression (Figure 7g–i), and exhibited significantly decreased level of Ki‐67 and increased level of γH2AX signals (Figure 7j–l). Taken together, these results demonstrated that enhanced RPA1 nuclear translocation mediated by ASCOR recruited and further enhanced DDX18 functions in promoting platinum resistance including genomic stability maintenance in vitro and in vivo.

### 2.7 ASCOR‐RPA1‐DDX18 Axis Promotes Platinum Resistance via Activating PI3K/Akt Activation Signaling

**FIGURE 8:**
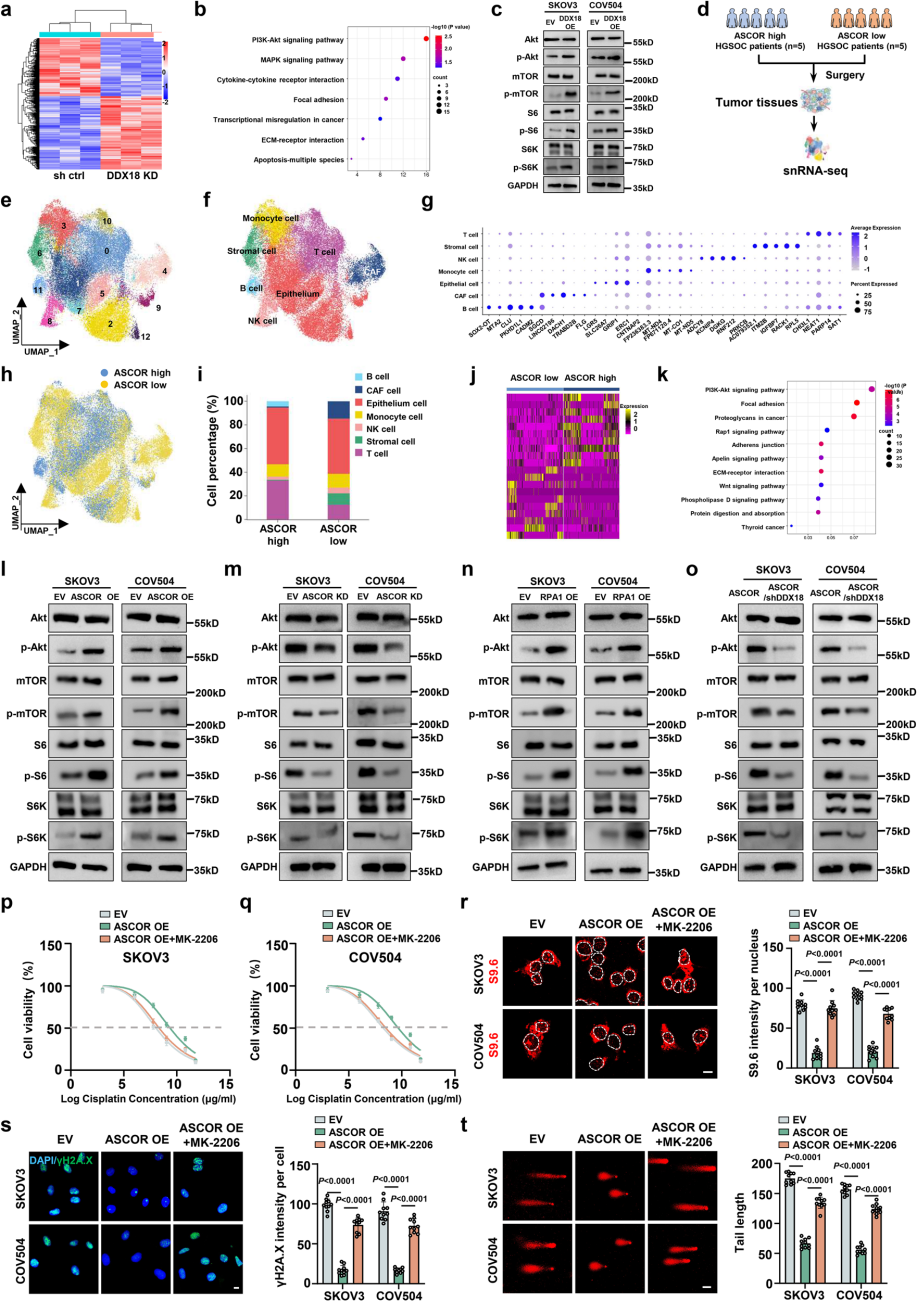
ASCOR‐RPA1‐DDX18 axis promotes platinum resistance via activating PI3K/Akt activation signaling. a) Heatmap of the differentially expressed mRNAs in RNA‐seq data upon DDX18 knockdown in SKOV3 cells. b) KEGG pathway enrichment analysis of differentially expressed genes from DDX18 knockdown RNA‐seq. c) Akt, P‐Akt, mTOR, p‐mTOR, S6, p‐S6, S6K, and p‐S6K were measured by western blot analysis upon DDX18‐overexpression in SKOV3 and COV504 cells. d) A schematic diagram illustrating the process of collecting and analyzing snRNA‐seq from five ASCOR‐high and five ASCOR‐low HGSOC tissues. e) A UMAP plot demonstrating 45159 cells grouped into 13 clusters. f) A UMAP plot demonstrating 45159 cells grouped into seven cell types. g) A dot plot demonstrating the expression of specific marker genes of each cell type. Dot size represented the percentage of marker gene expressed cells. Dot color represented the average expression level of marker genes. h) A UMAP plot demonstrating 45159 cells from 10 HGSOC patient tissues, colored by ASCOR levels of each patient. i) A column plot demonstrating the average proportion of different cell types in ASCOR high and low HGSOC tissues. j) Heatmap of the differentially expressed mRNAs in ASCOR‐high and ‐low epithelial cells. k) KEGG pathway enrichment analysis of upregulated gene expression in ASCOR high and low epithelial cells. l–n) Akt, P‐Akt, mTOR, p‐mTOR, S6, p‐S6, S6K and p‐S6K were measured by western blot analysis upon ASCOR‐overexpression (l), ASCOR knockdown (m), and RPA1‐overexpression (n) in SKOV3 and COV504 cells. o Western blot validation Akt and P‐Akt protein levels upon DDX18 knockdown in SKOV3 and COV504 ASCOR overexpression stable cells. p, q) Cell viability by CCK8 assay upon MK‐2206 in SKOV3 (p) and COV504 (q) ASCOR overexpression stable cells with cisplatin treatment at indicated concentrations for 36 h. IC50: SKOV3 control group, 6.551 µg/mL; SKOV3 ASCOR overexpression group, 8.867 µg/mL; SKOV3 ASCOR overexpression treatment with MK‐2206 group, 7.014 µg/mL; COV504 control group, 6.923 µg/mL; COV504 ASCOR overexpression group, 9.484 µg/mL; COV504 ASCOR overexpression treatment with MK‐2206 group, 7.324 µg/mL. r) Immunofluorescence (IF) staining of S9.6 upon MK‐2206 in SKOV3 and COV504 ASCOR overexpression stable cells, scale bar = 10 µm. s) IF staining of γH2A.X upon MK‐2206 in SKOV3 and COV504 ASCOR overexpression stable cells, scale bar = 10 µm. t) Comet assays upon MK‐2206 in SKOV3 and COV504 ASCOR overexpression stable cells, scale bar = 50 µm. shDDX18: DDX18 knockdown; OE: overexpression; EV: corresponding empty control. For r–t, samples were treated with cisplatin at 6 µg/mL. For c and l–t, data are from three independent experiments and shown as mean ± SD. *p* values are from unpaired two‐sided Student's *t*‐test. *p* < 0.05 was considered statistically significant.

To provide further insights for the ASCOR‐RPA1‐DDX18 axis in promoting HGSOC platinum resistance, RNA sequencing with DDX18‐knockdown SKOV3 cells was first performed (Figure 8a), with 578 downregulated and 179 upregulated mRNAs (Figure S7a), potentially linked to the functions of ASCOR‐RPA1‐DDX18 axis. KEGG pathway analysis of the differentially expressed genes upon DDX18 knockdown revealed that PI3K/Akt signaling pathway was the most significantly enriched in cancer‐associated pathway, suggesting that DDX18 may exert regulatory effects through this pathway (Figure 8b). Overexpression of DDX18 significantly elevated phosphorylated Akt (p‐Akt), phosphorylated mTOR (p‐mTOR), phosphorylated S6 (p‐S6) and phosphorylated S6K (p‐S6K) level in SKOV3 and COV504 cells (Figure 8c), indicating a potential positive regulatory relationship between the ASCOR‐RPA1‐DDX18 axis and the PI3K/Akt pathway.

To further characterize the ASCOR‐regulated TME and explore the downstream signaling that ASCOR influenced, we extended our investigations by conducting single‐nucleus transcriptome sequencing (snRNA‐seq) on 45,159 high‐quality cells from HGSOC specimens from patients with high (*n* = 5) and low (*n* = 5) ASCOR level (Figure 8d and Figure S7b). A total of 45,159 cells were retained and clustered into 13 distinct groups (Figure 8e). Based on the expression patterns of canonical cell‐type marker genes, cell clustering analysis identified seven major cell populations, including epithelial cells, cancer‐associated fibroblasts (CAFs), T cells, B cells, stromal cells, natural killer (NK) cells, and monocytes (Figure 8f,g). To characterize phenotypic heterogeneity associated with differential ASCOR expression, we re‐clustered the cells and overlaid their ASCOR expression groups (high vs. low) onto the clustering plot (Figure 8h). Consistent with our previous observations, epithelial cells constituted the dominant population in both groups (Figure 8i). Given that HGSOC is an epithelial‐derived malignancy, we focused subsequent analyses on epithelial cells. Subcluster analysis of the epithelial cell population revealed a significant upregulation of PI3K/Akt signaling especially in the high‐ASCOR subgroup (Figure 8j, k and Figure S7c). These findings from both DDX18 knockdown RNA‐seq and ASCOR expression‐stratified snRNA‐seq indicated that the PI3K/Akt signaling represents a key downstream signaling effector of the ASCOR‐RPA1‐DDX18 regulatory axis. On the basis of these observations, we then focused on investigating the downstream PI3K/Akt pathway in this context.

Overexpression of ASCOR significantly elevated p‐Akt, p‐mTOR, p‐S6 and p‐S6K levels, while its knockdown significantly decreased these levels; likewise, RPA1 overexpression also markedly increased p‐Akt, p‐mTOR, p‐S6 and p‐S6K levels, ASCOR without affecting total Akt, mTOR, S6 and S6K in SKOV3 and COV504 cells (Figure 8l–n), while DDX18 knockdown rescued ASCOR‐induced p‐Akt, p‐mTOR, p‐S6 and p‐S6K increase (Figure 8o). We also assessed the p‐Akt, p‐mTOR, p‐S6, and p‐S6K levels in the previous mice models (Figures 3a, g, t, g and S4 h). Consistently, IHC analysis of xenograft tumors demonstrated that p‐Akt, p‐mTOR, p‐S6, and p‐S6K levels were significantly increased in mouse tumors with sEV‐mediated ASCOR‐overexpression (Figures S8a and 3a–d), administration of sEVs from ASCOR‐high HGSOC patients (Figure S8b and Figure 3g–j), RPA1‐overexpression (Figures S9a and S4h–j), and DDX18‐overexpression (Figure S9b and Figure 6t–w) cohorts. The levels of p‐Akt, p‐mTOR, p‐S6, and p‐S6K were also rescued when DDX18 knockdown upon ASCOR overexpression in vivo (Figure S9c and Figure 7g–i). Treatment of MK2206, a selective allosteric inhibitor of Akt, was then employed to validate the involvement of the downstream PI3K/Akt signaling, in which MK‐2206 treatment significantly rescued ASCOR overexpression pro‐resistant phenotypes, with decreased proliferation capacity (Figure 8p,q), increased R‐loop accumulation (Figure 8r), and enhanced DNA damage (Figure 8s,t). Collectively, these data demonstrated that PI3K/Akt signaling is a downstream pathway of ASCOR‐RPA1‐DDX18 axis which was activated to promote platinum resistance.

### 2.8 ASO Targeting ASCOR Attenuates HGSOC Platinum Resistance In Vivo

**FIGURE 9:**
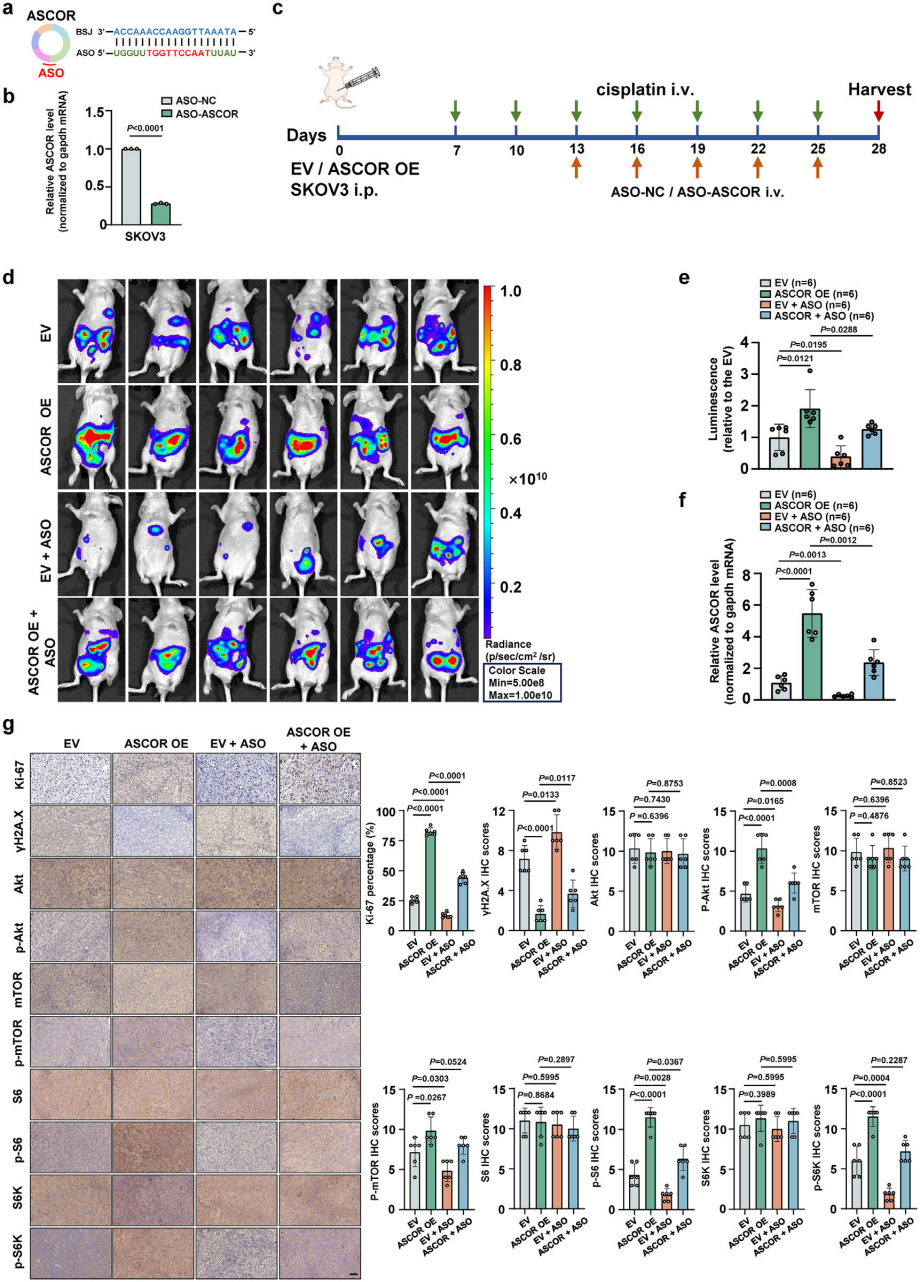
ASO targeting ASCOR attenuates high‐grade serous ovarian carcinoma (HGSOC) platinum resistance in vivo. a) Demonstration of the ASO sequences targeting the backsplicing junction of ASCOR. The central DNA sequences of the Gapmer ASO were marked red. b) RT‐qPCR analysis of ASCOR levels in SKOV3 cells after ASO‐mediated knockdown of ASCOR. c) Workflow of tumor‐bearing HGSOC mice model upon treatment with ASCOR ASO. d) Representative bioluminescent images of the xenograft intraperitoneal injection with ASCOR‐overexpression or control SKOV3 cells with or without treatment of ASO‐ASCOR. e) Quantification of bioluminescent imaging signal intensities. Mice were treated with 5 mg/kg cisplatin once every 3 days before the mice were sacrificed. f) Relative ASCOR levels in xenograft tumor were validated by RT‐qPCR. g) Immunohistochemical (IHC) analysis of Ki‐67, γH2A.X, Akt, P‐Akt, mTOR, p‐mTOR, S6, p‐S6, S6K and p‐S6K in the tumors dissected from each group, scale bar = 50 µm. The quantification of the markers in each group is shown with the bar figure. ASO: ASO‐mediated knockdown of ASCOR; NC: corresponding negative control; ASCOR OE: ASCOR overexpression; EV: corresponding empty control. For a and f‐g, data are from three independent experiments and shown as mean ± SD. *p* values are from unpaired two‐sided Student's *t*‐test. *p* < 0.05 was considered statistically significant.

Anti‐sense oligonucleotides (ASOs) are stable and potent agents for selective RNA targeting with high efficiency, which has been both validated in vitro and in vivo in functionality [54, 55, 56]. To evaluate the therapeutic potential of ASCOR inhibitor in HGSOC chemoresistance, we designed an ASO specifically targeting the BSJ of ASCOR with efficient knockdown of ASCOR in SKOV3 was validated (Figure 9a,b). SKOV3 stable cells with ASCOR overexpression and the control cells were intraperitoneally injected to the nude mice along with cisplatin treatment at Day 7, and ASO was treated starting from Day 13 (Figure 9c). Compared with empty control (EV) and ASCOR overexpression group, administration of ASCOR ASO significantly attenuated the platinum resistance with compromised tumor progression (Figure 9d,e). Levels of ASCOR with ASO treatment groups were validated markedly decreased (Figure 9f). ASO‐mediated ASCOR knockdown also led to decreased Ki‐67, p‐Akt, p‐mTOR, p‐S6, and p‐S6K levels and increased γH2AX levels, while total Akt, mTOR, S6 and S6K levels were unaffected (Figure 9g). Collectively, these results demonstrated that targeting ASCOR was a promising therapeutic strategy in HGSOC chemoresistance, as inhibition of ASCOR by ASO effectively attenuated the resistance.

## 3 Discussion

**FIGURE 10:**
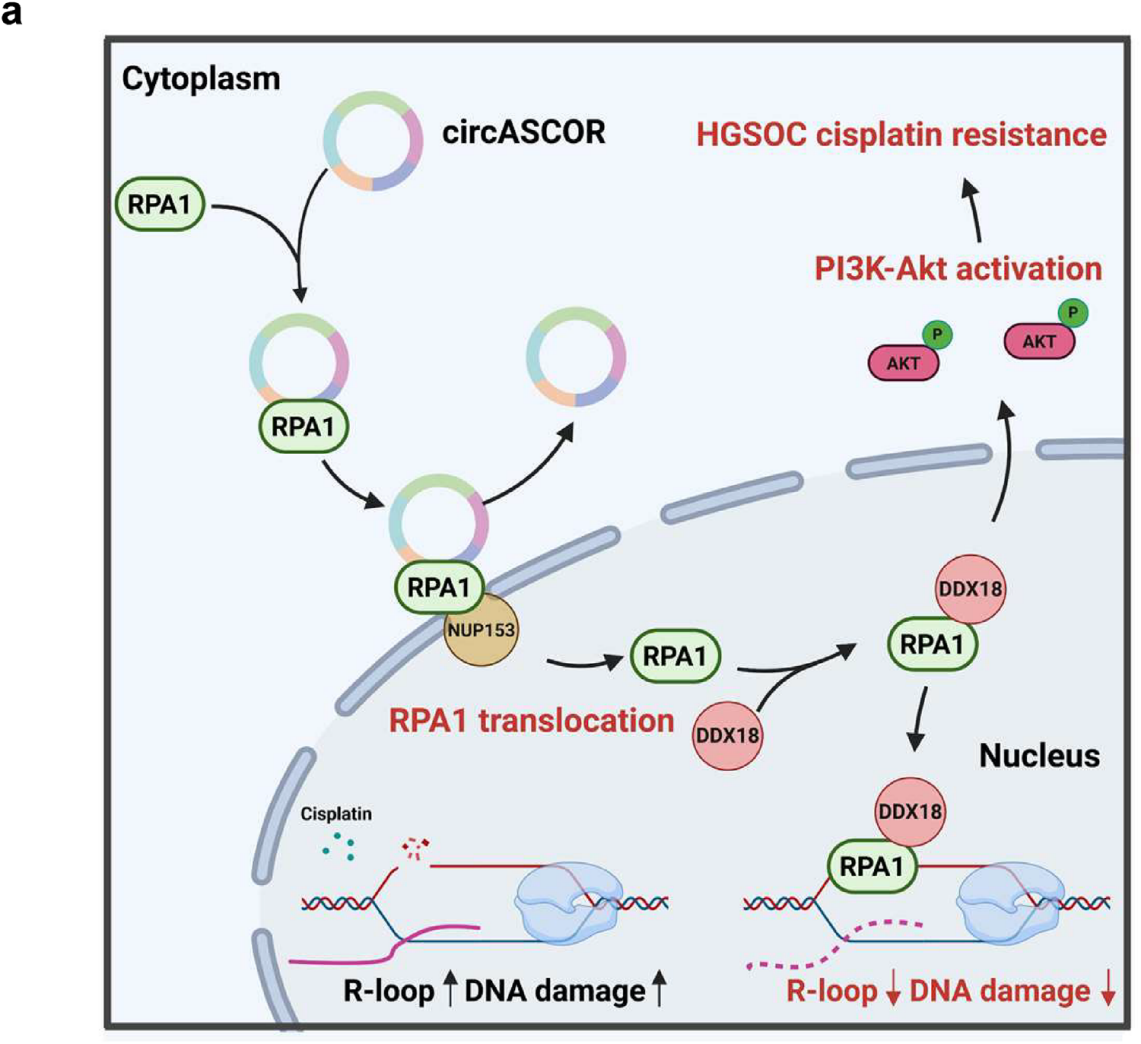
A proposed working model of ASCOR promotes high‐grade serous ovarian carcinoma (HGSOC) cisplatin resistance. a) ASCOR binds RPA1 and facilitates its NUP153‐dependent nuclear translocation, which recruits the RNA helicase DDX18, suppressing R‐loop formation and activates PI3K/Akt signaling to promote platinum resistance. This figure has been created with BioRender.com.

HGSOC remains the most lethal gynecologic malignancy, with advanced‐stage HGSOC often characterized by widespread peritoneal dissemination and malignant ascites, a hallmark of advanced cancer often associated with poor prognosis and chemotherapy resistance. Our focus on ascites and ascites‐derived sEVs in this study stems from both clinical relevance and biological significance for the reason that ascites serves as a unique tumor microenvironment (TME) niche, enriched with tumor cells, stromal cells, and bioactive molecules that collectively drive therapeutic phenotypes including drug resistance. Notably, ascites‐derived sEVs are the most informative composition within the ascites and have emerged as critical mediators of intercellular communication in the TME, encapsulating biomolecules such as circRNAs between cells. In multiple cancers, sEVs have been shown to shuttle oncogenic cargo to sensitive cells, inducing a persistent phenotype that confers drug resistance, such as initiating pre‐metastatic niche formation [57]. Our observation that ASCOR is abundant in ascites‐derived sEVs and correlates with chemotherapy resistance suggests that sEV‐associated ASCOR could serve as a noninvasive diagnostic marker for monitoring resistance development. Compared to tissue biopsies, ascites sampling is less invasive and can be repeated serially during treatment, enabling real‐time assessment of therapeutic response. By investigating ascites‐derived sEVs, we are able to not only capture a clinically targetable circRNA but also unveil a proof‐of‐concept mechanism by which drug resistance is propagated across the peritoneal cavity, offering a rationale for targeting sEV‐mediated signaling in late‐stage diseases (Figure 10a).

CircRNAs interacting with their protein counterparts have been recognized as one of the major functional mechanisms, yet their roles in modulating protein nuclear translocation remains poorly characterized. Circ‐Amotl1 is reported to promote nuclear retention of c‐myc, which in turn regulate a series of c‐myc targets in cancers [58]. Similarly, circRNA‐mTOR promotes the nuclear translocation of the RBP PC4 and SRSF1 interacting protein 1 (PSIP1) to increase lenvatinib resistance in hepatocellular carcinoma [59]. Our study further extends this phenomenon by demonstrating that ASCOR not only interacts with RPA1 to promote its nuclear translocation, but also showed that this process was facilitated by the nuclear pore complex protein NUP153. It is worth noting that the cytoplasmic ASCOR‐RPA1 interaction (Figure S3f–i), despite RPA1's predominant nuclear localization, reflects RPA1's trafficking dynamics. Synthesized in the cytoplasm, RPA1 undergoes transient cytoplasmic residency during its post‐translational maturation and nuclear import, which creates a window for ASCOR in engaging with RPA1 for translocation preparation. ASCOR binding to RPA1 in the cytoplasm enhances nuclear import machinery recognition or may prevent cytoplasmic sequestration, underscoring cytoplasmic regulation of nuclear protein function and emphasizing subcellular localization as a controlled process modulated by transient interactions by circRNAs. Moreover, the interaction and pro‐resistance effects could be transferred through sEVs for intercellular transmission, which enables the propagation of RPA1 nuclear translocation across cell populations, expanding the spatial scope of circRNA‐mediated protein regulation. Unlike linear RNAs, circRNAs are resistant to exonuclease degradation, making them stable cargo in sEVs—an attribute that likely enhances their ability to mediate long‐range signaling in the TME. However, the precise binding regions between ASCOR and RPA1, including nucleotide residues in ASCOR and binding motifs in RPA1, requires further identification via techniques such as iCLIP‐seq. It also remains unclear whether ASCOR‐RPA1 interaction is sufficient to drive nuclear translocation or if additional cofactors beyond NUP‐153 are involved. For example, does ASCOR‐RPA1 form a complex with known importins or other nuclear transport machinery to facilitate passage through the nuclear pore, representing another layer of regulation to explore.

RPA1, a multifunctional protein and key component of the RPA complex, is best known for its role in DNA replication and double‐strand break (DSB) repair, where it stabilizes single‐stranded DNA (ssDNA) generated during these processes and recruits repair machinery. Considering a large portion of ssDNA is generated by the transcription machinery, such as R loops [60, 61], DDX18‐induced R‐loop formation upon RPA1 nuclear translocation may provide insights into the associations of RPA1 and genome instability. Our study identifies a previously unrecognized role for RPA1, upon nuclear translocation facilitated by ASCOR, in interacting with the DEAD‐box helicase DDX18 to induce R‐loop formation and activate the PI3K‐Akt pathway, ultimately driving chemoresistance in HGSOC. This finding highlights RPA1's capacity to modulate signaling cascades independently of its canonical DNA repair functions. Nevertheless, it is also tempting to speculate that ASCOR‐RPA1 interactions in the cytoplasm indirectly influence RPA1's nuclear roles in DSB repair. For example, sequestration of RPA1 in the cytoplasm by ASCOR prior to nuclear translocation could transiently reduce its availability for DNA repair in the nucleus, potentially exacerbating genomic instability and creating a permissive environment for resistance development in an indirect, noncanonical manner.

We have provided lines of evidence that RPA1 interacted with DDX18 in the nucleus in the presence of ASCOR, which activated PI3K‐Akt pathway. Early studies demonstrated that RPA1 nuclear accumulation is essential for activating the ATR kinase, a master regulator of the DNA damage response [28]. More recently, RPA1 was shown to interact with transcription factors such as p53, modulating their transcriptional activity in response to genotoxic stress. It is interesting to explore further whether RPA1 form complexes with other traditional key nuclear proteins related to OC such as PARP1, BRCA1 to coordinate resistance phenotypes. It is also likely that other cellular pathways, such as DNA damage repair, p53‐dependent apoptosis, or other RPA1‐associated pathways, are impacted beyond PI3K‐Akt. R‐loops are a well‐recognized source of genomic instability [44] and previous study indicates that DDX18 affects R‐loop accumulation dynamics [53]. Recent study demonstrated that Akt1 facilitates R‐loop resolution via DHX9, and Akt inhibitor disrupts this interaction, impairing R‐loop resolution in HGSOC drug resistance [62]. However, the causal connection of DDX18, R‐loop to oncogenic signaling pathways such as PI3K‐Akt remains underexplored. Our data suggest that R‐loop accumulation coincides with PI3K‐Akt activation, raising the possibility that genomic stress triggered by R‐loops may feed into PI3K‐Akt signaling to promote cell survival. Whether DDX18‐induced R‐loops and PI3K‐Akt activation mechanistically linked or functionally parallel as a conserved mechanism across cancer types needs further investigation.

We have demonstrated the clinical significance of ASCOR‐RPA1‐DDX18 axis by ASOs targeting ASCOR to reverse chemoresistance. ASOs have shown efficacy in preclinical models of circRNA‐driven cancers. For example, ASOs targeting lncRNA CATED were recently shown to reduce chemoresistance in HGSOC by inhibiting MAPK pathway. With the advances of delivery systems such as lipid nanoparticles (LNPs) and virus‐like particles (VLPs) may also serve as leading platforms for circRNA therapeutics. Besides, either the use of a CATED inhibitor or blocking the ASCOR‐RPA1‐DDX18 axis could be an efficient measure to alleviate chemoresistance in HGSOC. Of note, while SKOV3 has been historically debated for its origin [46], it was selected as a model based on its derivation from ovarian adenocarcinoma ascites, which aligns with our focus on ascites‐derived sEVs, and critical cross‐validation of all cellular functional and mechanistic findings in COV504, an explicitly classified HGSOC cell line. We have also proved with evidence that sEV‐ASCOR from SKOV3 recapitulated the pro‐platinum resistance effects of clinical HGSOC ascites sEVs in vivo, confirming its biological relevance to HGSOC, demonstrating collectively that our conclusions are not cell line‐dependent and thus remain robust. In addition, despite our rescue experiments confirm the ASCOR‐RPA1‐DDX18 axis as the major mediator of sEV‐driven platinum resistance, it remains possible that other coexpressed components in ASCOR‐overexpressing cell‐derived sEVs contribute synergistically to the observed effects, which warrants further investigation of the comprehensive sEVs cargo profile in future studies.

Starting from screening with clinical HGSOC ascites collection, we have identified ASCOR as a key mediator of HGSOC chemoresistance, acting through promoting RPA1 nuclear translocation which triggers DDX18‐dependent R‐loop formation and ultimately PI3K‐Akt activation, with these effects amplified by sEV‐mediated intercellular transmission. Data from cultured cells and mice provide a rationale for developing diagnostic and therapeutic strategies targeting this axis, either alone or in combination with established or emerging therapies, ultimately aiming to improve outcomes for patients with HGSOC chemoresistance.

## 4 Materials and Methods

### 4.1 Clinical Samples

Clinical specimens were collected from patients with HGSOC at the First Affiliated Hospital of the University of Science and Technology of China between 2020 and 2023. All samples were collected during primary debulking surgery with no neoadjuvant chemotherapy allowed, and the platinum resistance status was determined retrospectively based on long‐term clinical follow‐up. Inclusion criteria were: (1) histopathologically confirmed HGSOC undergoing primary cytoreductive surgery; (2) FIGO 2014 stage II–IV; (3) no prior neoadjuvant chemotherapy or radiotherapy; (4) complete clinical records (including age, tumor grade, histopathology, surgical records, and follow‐up data); (5) survival follow‐up ≥12 months. Exclusion criteria included: (1) concurrent malignancies; (2) insufficient tissue or pathological data; (3) loss to follow‐up. Ascites collected during surgery was immediately processed for small extracellular vesicles (sEVs) extraction. Matched tumor tissues were snap‐frozen in liquid nitrogen; plasma samples were stored at −80°C. Ethical approval was obtained from the Research Ethics Committee of the First Affiliated Hospital of USTC (2021‐ky108), with written informed consent from all participants. Clinical information of patients with RNA‐seq in this study was presented in Table S1. Patients’ clinical information for qRT‐PCR validation in this study was presented in Tables S2 and S3. Clinical information of snRNA‐seq validation in this study was provided in Table S4.

### 4.2 Isolation and Identification of Small Extracellular Vesicles (sEVs)

Ascites and cell culture supernatants (50 mL) were centrifuged sequentially at 300 × *g* for 10 min (4°C; pellet discarded), 2000 × *g* for 20 min (debris removed), 20,000 × *g* for 60 min (apoptotic bodies removed), and twice at 100,000 × *g* for 70 min (Beckman Coulter L‐100XP). Pellets were resuspended in PBS and sterile‐filtered through a 0.22 µm filter. sEVs were characterized by Western blot for markers (TSG101, Hsp70, CD9, CD81, Alix) and absence of contaminants (Calnexin, GM130). For transmission electron microscopy (TEM), purified sEVs were adsorbed onto plasma‐treated hydrophilic copper grids (90 s), blotted to remove excess liquid, sequentially rinsed with 2% uranyl acetate, stained with 5 µL 2% uranyl acetate (90 s), blotted again, air‐dried, and imaged using TEM (FEI, USA). For nanoparticle tracking analysis (NTA), sEVs were resuspended in 0.22 µm‐filtered PBS. The NTA chamber (Particle Metrix, Germany) was rinsed with deionized water, calibrated with 100 nm polystyrene beads (1:250,000 dilution), flushed with 1×PBS, then injected with diluted sEVs suspensions for triplicate particle size distribution measurements.

### 4.3 Western Blotting

Proteins were extracted, quantified, and separated by 7.5–12% SDS‐PAGE, followed by transfer to polyvinylidene fluoride (PVDF) membranes. Membranes were blocked with 5% BSA‐TBST and incubated with primary antibodies overnight at 4°C. After washing, membranes were incubated with horseradish peroxidase (HRP)‐conjugated secondary antibodies at room temperature for 1 h. Signals were detected using ECL reagent (Ncmbiotech) and imaged on an ImageQuant LAS4000 (General Electric). Band densitometry was quantified using ImageJ software, with values normalized to GAPDH or Tubulin.

The following primary antibodies were used: Anti‐Alix (Proteintech, 12422‐1‐AP; 1:5000), Anti‐HSP70 (Proteintech, 10995‐1‐AP; 1:5000), Anti‐CD9 (Proteintech, 20597‐1‐AP; 1:5000), Anti‐CD81 (Proteintech, 27855‐1‐AP; 1:3000), Anti‐TSG101 (Abcam, ab125011; 1:1000), Anti‐Calnexin (Proteintech, 10427‐2‐AP; 1:5000), Anti‐GM130 (Proteintech, 11308‐1‐AP; 1:5000), Anti‐RPA1 (Abcam, ab79398; 1:1000), Anti‐DDX18 (Proteintech, 28502‐1‐AP; 1:1000), Anti‐H2AFY (Proteintech, 26875‐1‐AP; 1:500), Anti‐DDX3 (Proteintech, 11115‐1‐AP; 1:1000), Anti‐Histone H3 (Proteintech, 17168‐1‐AP; 1:2000), Anti‐NUP153 (Proteintech, 14189‐1‐AP; 1:1000), Anti‐Akt (Proteintech, 10176‐2‐AP; 1:1000), Anti‐PI3 Kinase p85 Alpha (Proteintech, 60225‐1‐Ig; 1:1000), Anti‐Phospho‐Akt (Ser473) (Proteintech, 66444‐1‐Ig; 1:1000), Anti‐mTOR (Proteintech, 66888‐1‐Ig; 1:5000), Anti‐Phospho‐mTOR (Ser2448) (Proteintech, 80596‐1‐RR; 1:5000), Anti‐S6 (Proteintech, 66886‐1‐Ig; 1:5000), Anti‐Phospho‐S6 (Ser235/236) (CST, 2211S; 1:1000), Anti‐S6k (Proteintech, 14485‐1‐AP; 1:2000), Anti‐Phospho‐S6K (Thr389/412) (Proteintech, 28735‐1‐AP; 1:1000), Anti‐HNRNPA2B1 (Proteintech, 14813‐1‐AP; 1:5000), Anti‐Tubulin (Proteintech, 10094‐1‐AP; 1:10,000), Anti‐GAPDH (Proteintech, 60004‐1‐Ig; 1:50,000), Anti‐S9.6 (Kerafast, ENH001; 1:200), Anti‐Phospho‐Histone H2A.X (Ser139) (CST, 80312S; 1:1000). Antibody validation data are available on the manufacturers' websites. Antibodies used in this study and Research Resource Identifiers (RRIDs) are provided in Table s5.

### 4.4 LIBRARY Construction, High‐Throughput Sequencing, and Bioinformatics Analysis

Total RNA was isolated from sEVs or cells using TRIzol reagent (Invitrogen) as described. RNA integrity and concentration were assessed by NanoDrop spectrophotometry and gel electrophoresis. For library preparation, rRNA was depleted using the Ribo‐Zero Gold Kit (Illumina) to enrich target RNAs. cDNA libraries were constructed by reverse transcription and sequenced on an Illumina platform (150 bp paired‐end reads). Sequencing reads were aligned to the GRCh38 reference genome using HISAT2 (v2.2.1) with default parameters. ENSG identifiers were converted to gene symbols for subsequent analysis. Differentially expressed genes were identified with significance thresholds of *p*‐value < 0.05 and | log_2_ (fold change) | ≥ 1. Heatmaps and volcano plots were generated using R software. Gene Ontology (GO) enrichment analysis was performed using Metascape with default parameters, and results were visualized using the ggplot2 package in R.

### 4.5 Cell Lines

The HEK293T (RRID: CVCL_0063) and ovarian cancer cell lines SKOV3 (RRID: CVCL_0532) and COV504 (RRID: CVCL_2424) were obtained from American Type Cancer Culture and CELLCOOK (Guangzhou, China), respectively. SKOV3 and COV504 were cultured in Dulbecco's Modified Eagle Medium (HyClone) supplemented with 10% fetal bovine serum (FBS; Gibco) and 1% penicillin‐streptomycin (100 U/mL) at 37°C in a humidified atmosphere with 5% CO_2_. Cells were passaged at 80% confluence using 0.25% trypsin‐EDTA (Gibco) for 2–3 min, followed by centrifugation at 1000 × *g* for 3 min. Mycoplasma contamination was monitored monthly by PCR and DAPI staining.

### 4.6 RNA Extraction

Cellular RNA was extracted using TRIzol reagent (Invitrogen). Briefly, 1 mL TRIzol was added per 10^6^ cells, homogenized, and phase‐separated with chloroform (1:5 v/v). RNA was precipitated with isopropanol, washed with 80% ethanol, and dissolved in RNase‐free water. For tumor tissue RNA extraction, samples were homogenized with Servicebio grinding beads and TRIzol until no visible debris remained, followed by the same extraction protocol.

### 4.7 Reverse Transcription

RNA concentration and purity (*A*_260_/*A*_280_ = 1.8–2.1) were assessed using a NanoDrop ND‐1000. Total RNA (1 µg) was reverse‐transcribed in a 20 µL reaction using HiScript III RT SuperMix (Abclonal) according to the manufacturer's instructions, with the following conditions: 50°C for 15 min, 85°C for 5 s.

### 4.8 Quantitative Real‐Time PCR (qRT‐PCR)

qRT‐PCR was performed in 15 µL reaction volumes on a Thermo Fisher instrument using the manufacturer's protocol, with cycling conditions: 95°C for 30 s; 40 cycles of 95°C for 5 s and 60°C for 30 s; followed by 60°C for 30 s and 20°C for 10 s. GAPDH served as the endogenous control. Relative expression was calculated using the 2(− ΔΔC_t_)method, normalized to calibrator samples. Primer sequences are listed in Table S6, and their efficiency was validated by standard curve analysis.

### 4.9 RNase R Treatment

Total RNA (1 µg) was incubated in a 50 µL reaction system containing 5 µL reaction buffer and 0.5 µL RNase R (Epicentre Technologies; experimental group) or DEPC‐water (negative control). Reactions were incubated at 37°C for 40 min, then diluted to 100 µL with DEPC‐water. Subsequently, 1/10 volume of sodium acetate and 2.5 volumes of absolute ethanol were added, and samples were precipitated at −80°C for 30 min. After centrifugation (12,000 × *g*, 30 min, 4°C), the supernatant was discarded, and pellets were washed twice with 70% ethanol, air‐dried, and dissolved in 20 µL DEPC‐water. Reverse transcription and qRT‐PCR were performed for both groups. circRNA stability was calculated as the relative expression ratio (RNase R‐treated / untreated).

### 4.10 Nucleocytoplasmic Separation

Cellular RNA compartmentalization was performed using confluent 10‐cm dishes under RNase‐free conditions. After trypsinization, cells were centrifuged (1000 × *g*, 3 min, room temperature) and washed twice with PBS. Pellets were resuspended in 1 mL ice‐cold lysis buffer (10 mM Tris‐HCl (pH 8.4), 0.5% NP‐40, 1.5 mM MgCl_2_, 140 mM NaCl, 1 mM DTT, 0.5% RNase inhibitor), gently vortexed, and centrifuged (1000 × *g*, 3 min, 4°C). The cytoplasmic fraction (supernatant) was collected, and 200 µL of this fraction was processed with 1 mL TRIzol for RNA extraction. The nuclear pellet was resuspended in 1 mL lysis buffer supplemented with 100 µL detergent solution (3.3% (w/v) sodium deoxycholate and 6.6% (v/v) Tween‐20), incubated on ice for 5 min, and centrifuged (1000 × *g*, 3 min, 4°C). After two additional washes with lysis buffer, the nuclear fraction was homogenized in 1 mL TRIzol supplemented with 50 µL lysis buffer for RNA isolation.

### 4.11 Fluorescence In Situ Hybridization (FISH)

Slide and cell preparation followed the immunofluorescence protocol, including fixation, permeabilization, and blocking. Blocking buffer was replaced with freshly prepared 2×SSC solution, and slides were completely immersed for 30 min at 37°C in a hybridization oven. Pre‐warmed hybridization buffer was equilibrated at 73°C for 30 min in a dry bath. Probes were thawed on ice, diluted to 1 µM in DEPC‐treated water, and denatured at 75°C for 10 min. Under light‐restricted conditions, denatured probes were mixed with streptavidin‐Cy3 (SA‐Cy3) at a 9:1 ratio and pre‐hybridized at 37°C for 30 min. This mixture was then combined with hybridization buffer at a 1:9 ratio. A humidity chamber was prepared using an RNase‐free tip box lined with DEPC‐treated water‐moistened filter paper and sealed with parafilm. Seventy‐five microliters of the probe mixture were applied to the parafilm, and slides were inverted from the 2×SSC solution onto the droplets (cell‐side down) for overnight hybridization (37°C, ≤16 h) in aluminum foil‐wrapped chambers. After hybridization, slides were washed sequentially: 4×SSC/0.1% Tween‐20 (37°C, 10 min), 2×SSC (60°C, 3 × 10 min), and 2×SSC (37°C, 3 × 10 min). Slides were mounted with 6–8 µL DAPI‐containing anti‐fade medium, gently air‐dried, and sealed with nail polish. Specimens were stored at 4°C protected from light until imaging with a Zeiss LSM880 confocal microscope (Airyscan detector). Probe sequences are listed in Table S6.

### 4.12 Culture of HGSOC Organoids

HGSOC primary cells were counted, and a cell suspension containing 4 × 10^5^ cells (approximately 400 µL) was prepared. An equal volume of Matrigel was added to the cell suspension, gently mixed, and kept on ice for later use. A 384‐well plate was used, with 8 µL of the mixture added to each well. The plate was then placed in a 37°C incubator with 5% CO_2_ for 45 min to allow Matrigel solidification. After incubation, 100 µL of complete culture medium was added to each well. The medium was refreshed with fresh culture medium every 3 days until organoid culture was completed.

### 4.13 Immunohistochemistry (IHC)

Formalin‐fixed paraffin‐embedded sections (4 µm) underwent heat‐induced antigen retrieval (60°C, 30 min), followed by dewaxing in xylene (2 × 10 min) and rehydration through a graded ethanol series (100%, 90%, 80%, 70%) and deionized water. Endogenous peroxidase activity was blocked with 3% H_2_O_2_ (10 min, room temperature), and sections were then blocked with 10% BSA (30 min, room temperature). Primary antibodies were applied overnight at 4°C: Ki67 (Proteintech, 27309‐1‐AP, 1:4000), Anti‐Phospho‐Histone H2A.X (Ser139) (CST, 80312S; 1:100), Akt (Proteintech, 10176‐2‐AP, 1:100), PI3 Kinase p85α (Proteintech, 60225‐1‐Ig, 1:1000), Phospho‐Akt (Ser473) (Proteintech, 66444‐1‐Ig, 1:100), mTOR (Proteintech, 66888‐1‐Ig, 1:1000), Phospho‐mTOR (Ser2448) (Proteintech, 80596‐1‐RR, 1:1000), Anti‐S6 (Proteintech, 66886‐1‐Ig; 1:500), Anti‐Phospho‐S6 (Ser235/236) (CST, 2211S; 1:300), Anti‐S6k (Proteintech, 14485‐1‐AP; 1:100), Anti‐Phospho‐S6K (Thr389/412) (Proteintech, 28735‐1‐AP; 1:100); antibody validation data are available on the manufacturer's website. After washing with PBS (3×), sections were incubated with secondary antibodies (30 min, room temperature). Antigen binding was visualized using strictly standardized DAB chromogen (manifesting as a brown signal), which minimized nonspecific background staining while maximally preserving the specificity and integrity of antigen‐antibody binding signals. The slides were subsequently counterstained with hematoxylin. Semi‐quantitative scoring combined staining intensity (0: none; 1: weak; 2: moderate; 3: strong) and percentage of positive cells (0: <1%; 1: 1–20%; 2: 21–50%; 3: 51–80%; 4: >80%). The immunoreactive score (IRS = intensity × percentage) ranged from 0 to 12. Specimens were categorized as: negative (IRS = 0), low (IRS 1–4), or high (IRS > 4). Scoring was performed by two independent, blinded histopathologists. Antibodies used in this study and Research Resource Identifiers (RRIDs) are provided in Table S5.

### 4.14 siRNA, Plasmid Construction, and Cell Transfection

siRNAs targeting RPA1 or DDX18 (GenePharma) were transfected at 6 µL (20 µM) per well in 6‐well plates using Lipofectamine 2000 (Invitrogen) according to the manufacturer's protocol. Briefly, 5 µL Lipofectamine 2000 was mixed with 125 µL Opti‐MEM (Gibco), and 6 µL siRNA was mixed with 125 µL Opti‐MEM. After incubating each mixture for 5 min, they were combined and incubated for an additional 20 min. Culture medium was removed, and cells were incubated with 750 µL Opti‐MEM; the transfection mixture was then added dropwise. After 6 h, the medium was replaced with complete culture medium. Knockdown efficiency was assessed by qRT‐PCR 24–48 h post‐transfection.

Plasmids encoding ASCOR, RPA1, site‐directed mutants, and protein truncation mutants were constructed by Miaoling Biotechnology (Wuhan, China). For lentiviral packaging, 293T cells were cotransfected with the plasmid constructs, psPAX2, and pMD2.G at a ratio of 4:3:1. Viral supernatants were collected 48 h post‐transfection, filtered through a 0.45 µm filter, and used to infect target cells (SKOV3, COV504) for 24 h. Stable cell lines were selected with 2–4 µg/mL puromycin for 14 days, and expression was validated by qRT‐PCR and Western blot. Sequences of siRNAs used in this study are provided in Table S7.

### 4.15 Cell Counting Kit‐8 (CCK8) Assay

Cells were seeded in triplicate in 96‐well plates at a density of 2 × 10^3^ cells/well and incubated overnight. After 24 h, cisplatin with indicated concentrations was added and plates were incubated for 36 h. CCK‐8 reagent (10 µL/well; NCM Biotech) was added, followed by incubation for 30 min at 37°C. Absorbance at 450 nm was measured using a Thermo Fisher Scientific microplate reader. Cell viability (%) was calculated as: (OD_450_ of treated group—OD_450_ of blank group) / (OD_450_ of control group—OD_450_ of blank group) × 100%. Data are presented as mean ± SD from three independent experiments. The Median inhibition concentration (IC50) values were calculated by nonlinear regression model using GraphPad Prism 9.

### 4.16 Colony‐Formation Assay

Cells were seeded at 500 cells/well in 6‐well plates and cultured for 14 days at 37°C in a humidified atmosphere with 5% CO_2_ in complete medium containing cisplatin or vehicle control. The medium was refreshed every 3 days. Colonies were fixed with 4% paraformaldehyde for 15 min, stained with 0.1% (w/v) crystal violet solution (Solarbio) for 20 min, and washed with PBS. Images were captured using a document scanner.

### 4.17 Mitochondrial Membrane Potential Assessment

The assay was performed according to the manufacturer's instructions for the Mitochondrial Membrane Potential Assay Kit (JC‐1; KeyGEN BioTECH, KGA1907‐100). Apoptosis‐induced cells in 12‐well plates were harvested by centrifugation (300 × *g*, 5 min), washed once with PBS, and incubated with JC‐1 working solution at 37°C in a 5% CO_2_ atmosphere for 15–20 min. After removing the solution, cells were washed twice with pre‐warmed 1× buffer, resuspended in culture medium, and immediately imaged using fluorescence microscopy or confocal microscopy. JC‐1 monomers (excitation/emission: 490/530 nm, green fluorescence) and aggregates (525/590 nm, red fluorescence) were detected: red fluorescence indicated healthy cells with intact membrane potential, while green fluorescence signaled early apoptosis due to membrane depolarization.

### 4.18 Immunofluorescence (IF)

Cells (2 × 10^5^ cells/well) were seeded in 6‐well plates containing coverslips and cultured to 50% confluency. After two washes with ice‐cold PBS, cells were fixed with chilled methanol:acetic acid (3:1, v/v) for 10 min. Following aspiration of the fixation buffer, cells were permeabilized twice with 0.5% Triton X‐100 in PBS (10 min per incubation on ice) and washed three times with PBST (0.1% Tween‐20) for 5 min per wash. Samples were blocked with 1% BSA for 30 min, then incubated with primary antibodies for 4 h at room temperature: anti‐RPA1 (Abcam, ab79398; 1:100) and anti‐Phospho‐H2A.X (Ser139) (CST, 2577S; 1:200). After three washes with PBST, Alexa Fluor 488/546‐conjugated secondary antibodies (Life Technologies; 1:200) were applied for 1 h at room temperature. Following additional washes, nuclei were counterstained with DAPI for 10 min, with three final rinses in PBST. Fluorescent images were acquired using a Zeiss LSM880 Airyscan confocal microscope. Antibody validation data are available on the manufacturers' websites. Antibodies used in this study and Research Resource Identifiers (RRIDs) are provided in Table S5.

### 4.19 Alkaline Comet Assay

Cells were washed with pre‐cooled PBS, centrifuged, and resuspended to a density of 1×10^6^ cells/mL. Gels were prepared in three layers with PBS. The first layer consisted of 100 µL of 1% normal melting point agarose (NMA), spread on slides, covered with a coverslip, and solidified at 4°C for 10 min. For the second layer, 10 µL of cell suspension (10^4^ cells) was mixed with 75 µL of melted 0.7% low melting point agarose (LMA; 60–80°C), applied to the first layer, covered with a coverslip, and solidified at 4°C for 10 min. After removing the coverslip, the third layer (75 µL of melted 0.7% LMA) was applied and solidified at 4°C. Slides were lysed in pre‐cooled lysis buffer (KeyGEN bioTECH) at 4°C for 1–2 h, rinsed three times with PBS, and immersed in alkaline electrophoresis buffer (300 mmol/L NaOH, 1 mmol/L EDTA, pH >10) for 20–60 min to unwind DNA. Electrophoresis was performed at 25 V for 20–30 min. Subsequently, slides were neutralized with Tris‐HCl (pH 7.5) and stained with PI or EB. Comet images were captured using a fluorescence microscope (excitation 515–560 nm).

### 4.20 sEVs Uptake Assay

sEVs were fluorescently labeled using the PKH67 kit (Umi biotech) according to the manufacturer's protocol. Briefly, PKH67 stock was diluted in Diluent C to 100 µM under light‐restricted conditions. sEVs (0.5–1 µg/µL in PBS) were incubated with 5 µM PKH67 in Diluent C at 4°C for 10 min. Unbound dye was removed by ultracentrifugation (100,000 × *g*, 4°C, 70 min) with PBS washing, and labeled sEVs were resuspended in 200 µL PBS. These were cocultured with pre‐seeded cell monolayers for 24 h under light‐restricted conditions. Cell slides were then processed for FISH analysis as previously described.

### 4.21 EdU Proliferation Assay

Cell proliferation was assessed using the BeyoClick EdU‐488 Kit (KeyGEN BioTECH) according to the manufacturer's instructions. Logarithmic‐phase cells were trypsinized, resuspended in complete medium, and seeded in 12‐well plates at 2–5 × 10^4^ cells/well (triplicate wells per group). After overnight attachment, cells were treated with drug‐containing medium for 48 h under standard culture conditions. For EdU labeling, cells were incubated with pre‐warmed EdU working solution (10 mM in complete medium) for 2 h. Cells were then fixed with 4% paraformaldehyde, permeabilized with 0.3% Triton X‐100, and incubated with freshly prepared Click Reaction mixture. Following washes, nuclei were counterstained with DAPI for 10 min and rinsed three times with PBST. Fluorescent images were acquired using an inverted microscope.

### 4.22 In Vivo Xenograft Experiments

The animal experiments were approved by the Animal Care and Use Committee of the First Affiliated Hospital of USTC (2025‐N(A)‐0105). Stable overexpressing SKOV3 cells, control cells, and wild‐type SKOV3 cells were expanded in culture, transduced with luciferase‐expressing lentivirus, and resuspended at 5 × 10^7^ cells/mL. Five‐week‐old female BALB/c nude mice were maintained under SPF conditions and received intraperitoneal injections of 5 × 10^6^ cells per mouse to establish peritoneal tumor models. For sEVs modulation studies, mice bearing wild‐type SKOV3 tumors were randomized 1 week post‐inoculation into groups receiving intravenous cisplatin (5 mg/kg) or ASCOR‐ASO every 3 days,&&&& combined with intraperitoneal injections of either: (1) ASCOR‐overexpressing sEVs (sEV‐OE), control sEVs (sEV‐EV), or PBS; or (2) sEVs from ASCOR‐low HGSOC ascites (sEV‐ASCOR low), sEVs from ASCOR‐high HGSOC ascites (sEV‐ASCOR high), versus PBS. Parallel studies evaluated RPA1‐overexpressing, DDX18‐overexpressing, shDDX18 in RPA1‐overexpressing, and corresponding empty control SKOV3 cells (*n* = 6/group) using identical cisplatin dosing initiated 1 week post‐engraftment. Tumor progression was monitored via weekly bioluminescence imaging after intraperitoneal D‐luciferin administration (50 mg/kg), with terminal analysis performed 3 weeks after treatment initiation. Collected tumors were subjected to histopathological and molecular characterization.

### 4.23 RNA Pull‐Down Assay

1×10^7^ PBS‐washed cells were UV‐crosslinked (254 nm, 1200 mJ/cm^2^, 2 min) and lysed in buffer containing 50 mM Tris‐HCl (pH 8.0), 150 mM NaCl, 5 mM EDTA, 10% NP‐40, 10% SDS, 100× protease/phosphatase inhibitors, 2 mM DTT, and RNase inhibitor. Lysates were incubated with 5′‐biotinylated antisense probes targeting ASCOR or scrambled control oligos (50 pmol/sample) for 4 h at room temperature with rotation (20 rpm), followed by capture with pre‐washed Dynabeads M‐280 (100 µL/sample; Invitrogen) for 1 h. Magnetically isolated complexes were washed sequentially: twice with native lysis buffer and three times with high‐stringency buffer (lysis buffer supplemented with 500 mM NaCl). Captured complexes were split for parallel analysis: RNA was extracted via digestion with DNase I (37°C, 20 min) and Proteinase K (30 µg, 56°C, 20 min), followed by TRIzol purification and RT‐qPCR; proteins were separated by SDS‐PAGE and immunoblotted with anti‐RPA1 (Invitrogen, PA5‐99469; 1:1000). Probe sequences are listed in Table S6.

### 4.24 Protein Silver Staining and Mass Spectrometry Analysis

Protein gel silver staining was performed using the Sangon Biotech Rapid Silver Staining Kit (C500021). Following SDS‐PAGE, gels were transferred to 10 cm petri dishes, with stacking gels and empty lanes removed. Gels were fixed in 40% ethanol/10% acetic acid (20 mL) with gentle agitation for 15 min per wash, repeated three times. Sensitization was performed with 20 mL 1× sensitization solution for 45 min, followed by three 5‐min washes with ddH_2_O. Staining solution (4 mL ethanol + 4 mL 5× stain + 12 mL ddH_2_O) was applied for 30 min with shaking, then rinsed twice in ddH_2_O (30 s per wash). Development was initiated by adding 20 mL Developer A supplemented with 11 µL Developer B, with continuous agitation until bands reached optimal intensity. Reactions were terminated with 20 mL 1× stop solution for 15 min, followed by three 5‐min washes in ddH_2_O. For mass spectrometry, gels were treated sequentially with 10 mL Silver Removal Solution A and 10 mL Solution B (10–60 min with shaking), then washed three times in ddH_2_O for 10 min each. Protein bands of interest were documented, excised, and processed for mass spectrometry analysis.

### 4.25 RNA Immunoprecipitation (RIP) Assay

RNA‐protein complexes were stabilized by UV crosslinking (254 nm, 1200 mJ/cm^2^, 2 min). Crosslinked cells were lysed in RIPA buffer (50 mM Tris‐HCl (pH 8.0), 150 mM NaCl, 5 mM EDTA, 10% NP‐40, 10% SDS, 100× protease/phosphatase inhibitor cocktail, 2 mM DTT, RNase inhibitor) and sonicated (3 s pulse/6 s pause cycles, 5 min, 30% amplitude). Lysates were clarified by centrifugation (12,000 × *g*, 15 min, 4°C), then incubated overnight at 4°C with Dynabeads Protein G (Invitrogen) pre‐conjugated with 5 µg of anti‐RPA1 (Abcam, ab79398), anti‐DDX18 (Proteintech, 28502‐1‐AP), anti‐HNRNPA2B1 (Proteintech, 14813‐1‐AP), anti‐FLAG (Proteintech, 20543‐1‐AP), or control IgG. Beads were washed three times with high‐salt buffer (500 mM NaCl). Immunoprecipitated material was split into two parts: one half underwent sequential digestion with DNase I (37°C, 20 min) and Proteinase K (30 µg, 56°C, 20 min) for TRIzol‐based RNA isolation and RT‐qPCR analysis; the other half was processed for immunoblotting to verify target enrichment. Antibodies used in this study and Research Resource Identifiers (RRIDs) are provided in Table s5.

### 4.26 Co‐Immunoprecipitation (co‐IP) Assay

Protein–protein complexes were stabilized by UV cross‐linking (254 nm, 1200 mJ/cm^2^, 2 min). Cross‐linked cells were lysed in RIPA buffer (50 mM Tris‐HCl (pH 8.0), 150 mM NaCl, 5 mM EDTA, 10% NP‐40, 10% SDS, 100× protease/phosphatase inhibitor cocktail, 2 mM DTT) and sonicated (3 s pulse/6 s pause, 5 min, 30% amplitude). Lysates were clarified by centrifugation (12,000 × *g*, 15 min, 4°C), then incubated overnight at 4°C with 2 µg of anti‐RPA1 (Abcam, ab79398), anti‐DDX18 (Proteintech, 28502‐1‐AP), or control IgG. Protein G Dynabeads (100 µL; Invitrogen) were added to capture antibody complexes during overnight incubation (4°C, gentle rotation). Beads were washed three times with RIPA buffer, and interactions were validated by Western blot. Antibodies used in this study and Research Resource Identifiers (RRIDs) are provided in Table S5.

### 4.27 Progression‐Free Survival Analysis

All patients were followed up throughout the entire period. The expression levels of ASCOR, RPA1 and DDX18 in clinical samples were detected separately, and patients were divided into quartile‐based groups: low levels (first quartile), intermediate levels (second and third quartiles), and high levels (fourth quartile) according to the expression levels. Survival curves were plotted based on the follow‐up data using GraphPad Prism 9.

### 4.28 Statistical Analysis

Data were analyzed using GraphPad Prism 9. Normality was assessed via the Shapiro‐Wilk test. Parametric data were analyzed using Student's unpaired *t*‐test (two groups) or one‐way ANOVA with Tukey's post‐hoc test (≥3 groups). Nonparametric data were analyzed using the Kruskal–Wallis test with Dunn's post‐hoc correction. Survival curves were generated using the Kaplan–Meier method and compared with the log‐rank test. Correlations were evaluated using Pearson's (parametric) or Spearman's (nonparametric) tests based on data distribution. All experiments included ≥3 biological replicates. Parametric data are presented as mean ± SD, and nonparametric data as median with interquartile range (IQR).

## Author Contributions

H.L. performed conceptualization, data curation, formal analysis, funding acquisition, investigation, methodology, validation, visualization, writing – original draft, writing – review and editing. C.Z. worked on conceptualization, data curation, formal analysis, investigation, methodology, writing – original draft, writing – review and editing. X.Y. worked on formal analysis, methodology, visualization, writing – review and editing. Y.L. focused on formal analysis, writing – review and editing. J.Z. performed formal analysis, writing – review and editing. Y.L. worked on formal analysis, writing – review and editing. H.X. performed formal analysis, writing – review and editing. C.P. worked on conceptualization, investigation, supervision, writing – original draft, writing – review and editing. G.S. worked on conceptualization, funding acquisition, supervision, writing – original draft, writing – review and editing. L.C. worked on conceptualization, funding acquisition, supervision, writing – original draft, writing – review and editing. Y.Z. worked on conceptualization, data curation, formal analysis, funding acquisition, investigation, methodology, project administration, resources, supervision, writing – original draft, writing – review and editing.

## Conflicts of Interest

The authors declare no conflict of interest. Y.Z., L.C., G.S. and C.P. have an ownership interest in a patent related to this research.

## Supporting information




**Supporting File**: advs74359‐sup‐0001‐SuppMat.docx.

**Supporting File**: advs74359‐sup‐0002‐Figure S1‐S9.

**Supporting File**: advs74359‐sup‐0003‐ Table S1‐S7.docx.

## Data Availability

All original RNA‐seq reported in this paper are publicly available through GSE247337, GSE313584 and GSE315520. All study data and materials are included in the article and/or supporting information. The data that support the findings of this study are available from the corresponding author upon reasonable request.
