# Supplementary material for: Ascites circRNA ASCOR Drives Platinum Resistance of High‐Grade Serous Ovarian Cancer by Facilitating RPA1 Nuclear Translocation
Source: Adv Sci (Weinh). 2026 Feb 12;13(23):e18922. doi: 10.1002/advs.202518922 (PMC13104110; doi:10.1002/advs.202518922)
Supplement: Supplementary file 1 — Supporting File: advs74359‐sup‐0001‐SuppMat.docx. [file ADVS-13-e18922-s003.docx]

**Supplementary figure legends**

**Supplementary figure 1.** **Characterization of the isolated ascites sEVs from HGSOC patients.** **a**, **b** Representation images and size analysis of isolated sEVs from ascites by using Transmission Electron Microscopy (TEM) (**a**) and Nanoparticle Tracking Analysis (NTA) (**b**), scale bar = 200 nm. S: sensitive; R: resistant. **c** Western blot validation protein level of Alix, Hsp70, TSG101, CD9, CD81, GM130 and Calnexin. **d** Levels of ASCOR in ascites sEVs, primary tumor, omentum and peritoneum tissues from HGSOC patients (n=11). **e** RT-qPCR validation for the ASCOR overexpression in KEK293T cells. **f** RT-qPCR validation for ASCOR overexpression in sEVs of donor cells. **g, h** Western blot validation of HNRNPA2B1 RIP (**g**) and relative enrichment of ASCOR (**h**) in SKOV3 and COV504 cells. **i** Relative protein levels examined by Western blot upon HNRNPA2B1 knockdown in SKOV3 and COV504 cells. **j** RT-qPCR validation for ASCOR overexpression in sEVs of HNRNPA2B1 knockdown in SKOV3 and COV504 donor cells. **k** RPRD1A mRNA examined by RT-qPCR in ASCOR-overexpressing or control SKOV3 and COV504 cells. ASCOR OE: ASCOR overexpression; EV: corresponding empty control. For **c**-**k,** data are from three independent experiments and shown as mean ± SD. *P* values are from unpaired two-sided Student’s *t*-test. *P* < 0.05 was considered statistically significant.

**Supplementary figure 2. ASCOR inhibits DNA damage *in vitro*.** **a** Western blot validation protein level of γH2A.X in ASCOR-overexpressing or control SKOV3 and COV504 cells with cisplatin treatment. **b** Western blot validation protein level of γH2A.X upon pre-incubated with sEVs derived from ASCOR-overexpressing or control SKOV3 and COV504 cells with cisplatin treatment. For **a**-**b**, samples were treated with cisplatin at 6 μg/ml. For **a**-**b,** data are from three independent experiments, ASCOR OE: ASCOR overexpression; EV: corresponding empty control.

**Supplementary figure 3. ASCOR interacts with RPA1 protein. a** Western blot validation of AGO2 RIP and relative pulldown efficiency of ASCOR in SKOV3 and COV504 cells. **b** Top predicted candidates that directly interacted with ASCOR in SKOV3. **c** Collision-induced dissociation mass spectrum showing the structural diagram of RPA1 protein. **d**, **e** Overall survival (OS) (**d**) and progression-free survival (PFS) (**e**) curve of RPA1 in ovarian cancer patients. **f** Progression-free survival (PFS) analysis of the HGSOC patients was performed in the RPA1-high/intermediate/low tissues group. RPA1 high vs. RPA1 intermediate, P = 0.0267; RPA1 high vs. RPA1 low, P = 0.0449; RPA1 intermediate vs. RPA1 low, P = 0.6269. **g**, **h** The pulldown efficiency of ASCOR (**g**) and Western blot validation (**h**) of RPA1 in ASCOR pulled-down proteins in the cytoplasm of SKOV3 and COV504 cells were presented. **i**, **j** Western blot validation of RPA1 RIP (**i**) and relative enrichment of ASCOR (**j**) in the cytoplasm of SKOV3 and COV504 cells. **k**, **l** Relative RPA1 mRNA and protein level examined by RT-qPCR (**k**) and Western blot (**l**) in RPA1-overexpressing or control SKOV3 and COV504 cells. For **a** and **f**-**i,** data are from three independent experiments and shown as mean ± SD. *P* values are from unpaired two-sided Student’s *t*-test. *P* < 0.05 was considered statistically significant.

**Supplementary figure 4. RPA1 promoting HGSOC cisplatin resistance.** **a**, **b** Cell viability assessed by CCK8 assay upon RPA1 overexpression in SKOV3 (**a**) and COV504 (**b**) cells with cisplatin treatment at indicated concentrations for 36 hours. IC50: SKOV3 control group, 5.372 μg/ml; SKOV3 RPA1 overexpression group, 8.574 μg/ml; COV504 control group, 5.955 μg/ml; COV504 RPA1 overexpression group, 8.991 μg/ml. **c** Colony formation assay upon RPA1-overexpression in SKOV3 and COV504 cells. **d** EdU assays upon RPA1-overexpression in SKOV3 and COV504 cells, scale bar = 50 μm. **e** JC-1 staining for mitochondrial membrane potential measurement upon RPA1-overexpression in SKOV3 and COV504 cells, scale bar = 20 μm. **f** IF staining of γH2A.X upon RPA1-overexpressing or control SKOV3 and COV504 cells, scale bar = 10 μm. **g** Comet assay upon RPA1-overexpressing or control SKOV3 and COV504 cells, scale bar = 50 μm. **h**, **i** Representative bioluminescent images of the xenograft intraperitoneal injection with RPA1-overexpressing or control SKOV3 cells (**h**) and quantification of bioluminescent imaging signal intensities (**i**) after 4 weeks. Mice were treated with 5 mg/kg cisplatin once every 3 days before the mice were sacrificed. **j** Relative RPA1 mRNA levels in xenograft tumor were detected by RT-qPCR. **k** IHC analysis and quantification of Ki-67 and γH2A.X upon the tumors dissected from each group, scale bar = 50 μm. The quantification of the markers in each group is shown in the bar figure. **l**, **m** Cell viability by CCK8 assay upon RPA1 WT or Del_1 overexpression in SKOV3 (**l**) and COV504 (**m**) cells with cisplatin treatment. IC50: SKOV3 control group, 6.084 μg/ml; SKOV3 RPA1 overexpression group, 9.165 μg/ml; SKOV3 RPA1 Del_1 overexpression group, 6.377 μg/ml; COV504 control group, 6.750 μg/ml; COV504 RPA1 overexpression group, 9.183 μg/ml; COV504 RPA1 Del_1 overexpression group, 7.185 μg/ml. For **c**-**g**, samples were treated with cisplatin at 6 μg/ml. RPA1 OE: RPA1 overexpression; Del_1 OE: RPA1-Del_1 overexpression; EV: corresponding empty control. For **A**-**O,** data are from three independent experiments and shown as mean ± SD. *P* values are from unpaired two-sided Student’s *t*-test. *P* < 0.05 was considered statistically significant.

**Supplementary figure 5. Identification of NUP153 as a nuclear translocation-related protein**. **a** The Venn diagram illustrates the intersection between literature-derived 46 nuclear translocation-related proteins (Yang Y, et al. 2023) and 221 mass spectrometry-identified interacting proteins, with NUP153 highlighted as a key overlapping candidate. **b** Collision-induced dissociation mass spectrum showing the structural diagram of NUP153 protein.

**Supplementary figure 6.** **Identification of DDX18 interacting with RPA1**. **a** Top predicted candidates that directly interacted with RPA1 in SKOV3 cells. **b** Collision-induced dissociation mass spectrum showing the structural diagram of DDX18 protein. **c** Neither of H2AFY or DDX3 was detected in RPA1 co-IP of the SKOV3 and COV504 cells using anti-RPA1 antibody. **d**, **e** OS (**d**) and PFS (**e**) curve of DDX18 in ovarian cancer patients. For **c,** data are from three independent experiments. **f** Progression-free survival (PFS) analysis of the HGSOC patients was performed in the DDX18-high/intermediate/low tissues group. DDX18 high vs. DDX18 intermediate, P = 0.0357; DDX18 high vs. DDX18 low, P = 0.0337; DDX18 intermediate vs. DDX18 low, P = 0.6202. For **f,** data are from three independent experiments and shown as mean ± SD. *P* values are from unpaired two-sided Student’s *t*-test. *P* < 0.05 was considered statistically significant.

**Supplementary figure 7.** **Data of RNA-seq and snRNA-seq. a** Volcano plot of the differentially expressed mRNAs in RNA-seq data of DDX18 knockdown and negative control SKOV3 cells. **b** Relative ASCOR level examined by RT-qPCR in HGSOC tissues for snRNA-seq. **c** Volcano plot of the differentially expressed mRNAs in ASCOR high and low epithelial cells. For **b,** data are from three independent experiments and shown as mean ± SD. *P* values are from unpaired two-sided Student’s *t*-test. *P* < 0.05 was considered statistically significant.

**Supplementary figure 8.** **ASCOR promoting HGSOC cisplatin resistance via PI3K/Akt pathway. a** IHC analysis of Akt, P-Akt, mTOR, p-mTOR, S6, p-S6, S6K and p-S6K upon the tumors dissected from intraperitoneal injection with SKOV3 WT cells treated with sEVs-mediated ASCOR-overexpressing or control, scale bar = 50 μm. **b** IHC analysis of Akt, P-Akt, mTOR, p-mTOR, S6, p-S6, S6K and p-S6K upon the tumors dissected from intraperitoneal injection with SKOV3 WT cells treated with sEVs-ASCOR low or sEVs-ASCOR high derived from HGSOC ascites, scale bar = 50 μm. sEV-OE: sEVs-mediated ASCOR overexpression; sEV-EV: sEVs-mediated corresponding empty control, sEV-ASCOR low: sEVs from ASCOR-low HGSOC ascites; sEV-ASCOR high: sEVs from ASCOR-high HGSOC ascites. For **a**-**b,** the quantification of the Akt, P-Akt, mTOR, p-mTOR, S6, p-S6, S6K and p-S6K in each group is shown in the bar figure**,** data are from three independent experiments and shown as mean ± SD. *P* values are from unpaired two-sided Student’s *t*-test. *P* < 0.05 was considered statistically significant.

**Supplementary figure 9.** **ASCOR promoting HGSOC cisplatin resistance via RPA1/DDX18 and regulation of the PI3K/Akt pathway. a** IHC analysis of Akt, P-Akt, mTOR, p-mTOR, S6, p-S6, S6K and p-S6K upon the tumors dissected from intraperitoneal injection with RPA1-overexpressing or control SKOV3 cells, scale bar = 50 μm. **b** IHC analysis of Akt, P-Akt, mTOR, p-mTOR, S6, p-S6, S6K and p-S6K upon the tumors dissected from intraperitoneal injection with DDX18-overexpressing or control SKOV3 cells, scale bar = 50 μm. **c** IHC analysis of Akt, P-Akt, mTOR, p-mTOR, S6, p-S6, S6K and p-S6K upon the tumors dissected from intraperitoneal injection with SKOV3 cells stably overexpressing ASCOR with or without DDX18 knockdown, as well as control SKOV3 cells, scale bar = 50 μm. RPA1 OE: RPA1 overexpression; DDX18 OE: DDX18 overexpression; ASCOR OE: ASCOR overexpression; ASCOR/shDDX18: DDX18 knockdown in cells stably overexpressing ASCOR; EV: corresponding empty control. For **a**-**c,** the quantification of the Akt, P-Akt, mTOR, p-mTOR, S6, p-S6, S6K and p-S6K in each group is shown in the bar figure**,** data are from three independent experiments and shown as mean ± SD. *P* values are from unpaired two-sided Student’s *t*-test. *P* < 0.05 was considered statistically significant.
