# Supplementary material for: Ascites circRNA ASCOR Drives Platinum Resistance of High‐Grade Serous Ovarian Cancer by Facilitating RPA1 Nuclear Translocation
Source: Adv Sci (Weinh). 2026 Feb 12;13(23):e18922. doi: 10.1002/advs.202518922 (PMC13104110; doi:10.1002/advs.202518922)

Supplementary figure 1

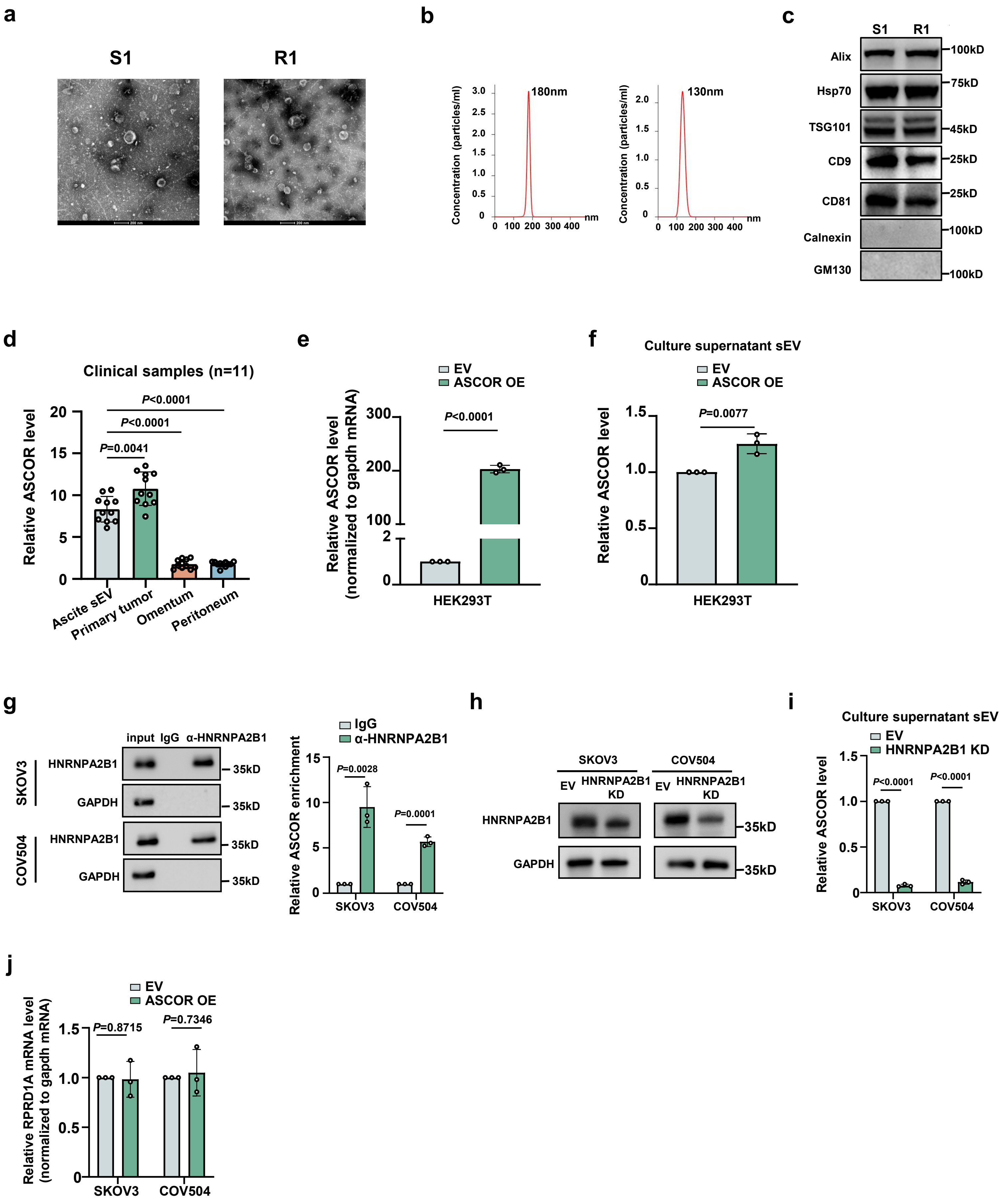

Supplementary figure 2

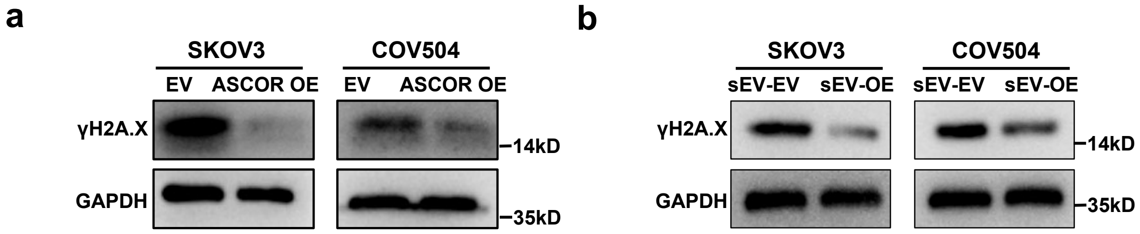

Supplementary figure 3

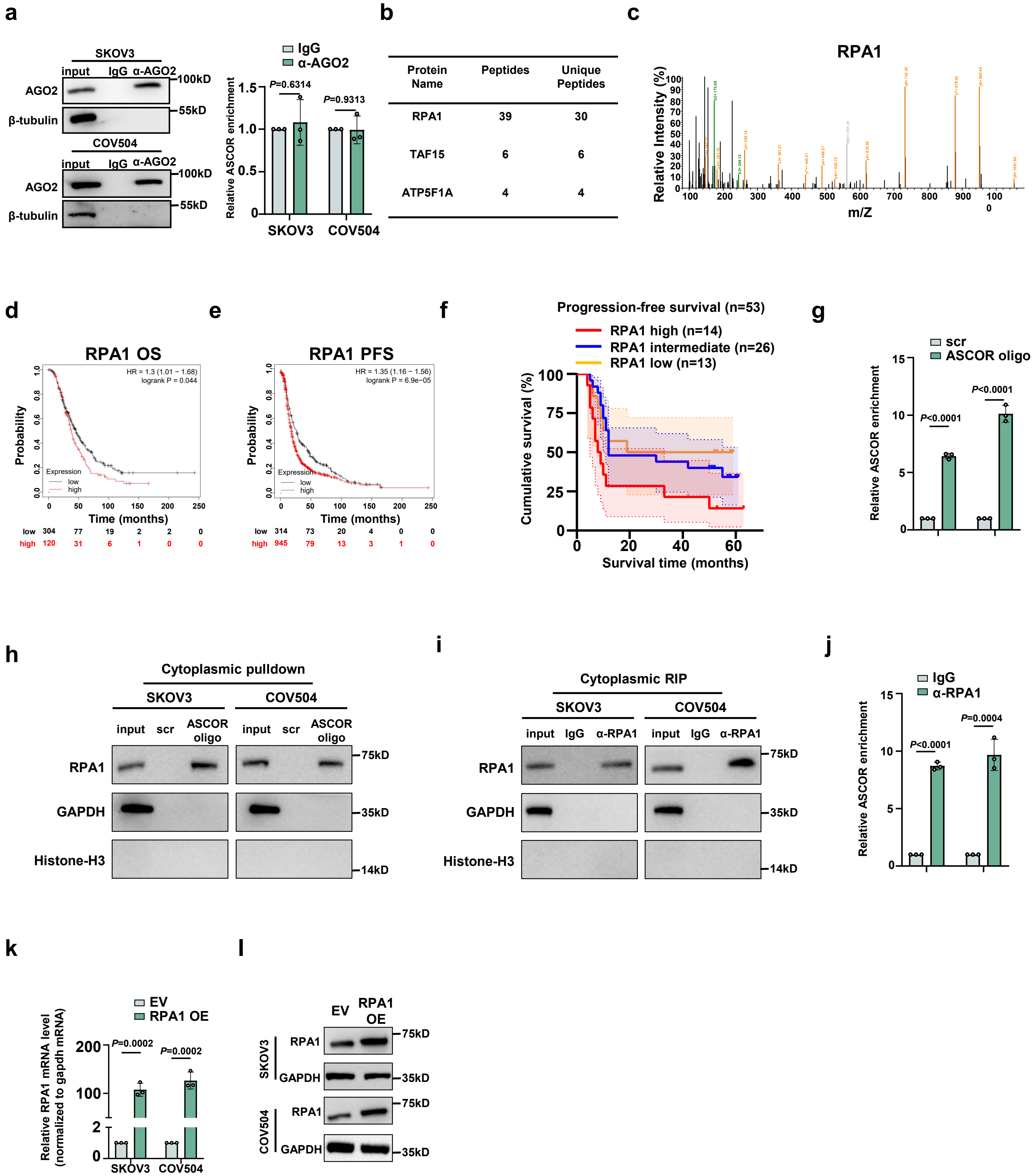

Protein Name	Peptides	Unique Peptides
RPA1	39	30
TAF15	6	6
ATP5F1A	4	4

Supplementary figure 4

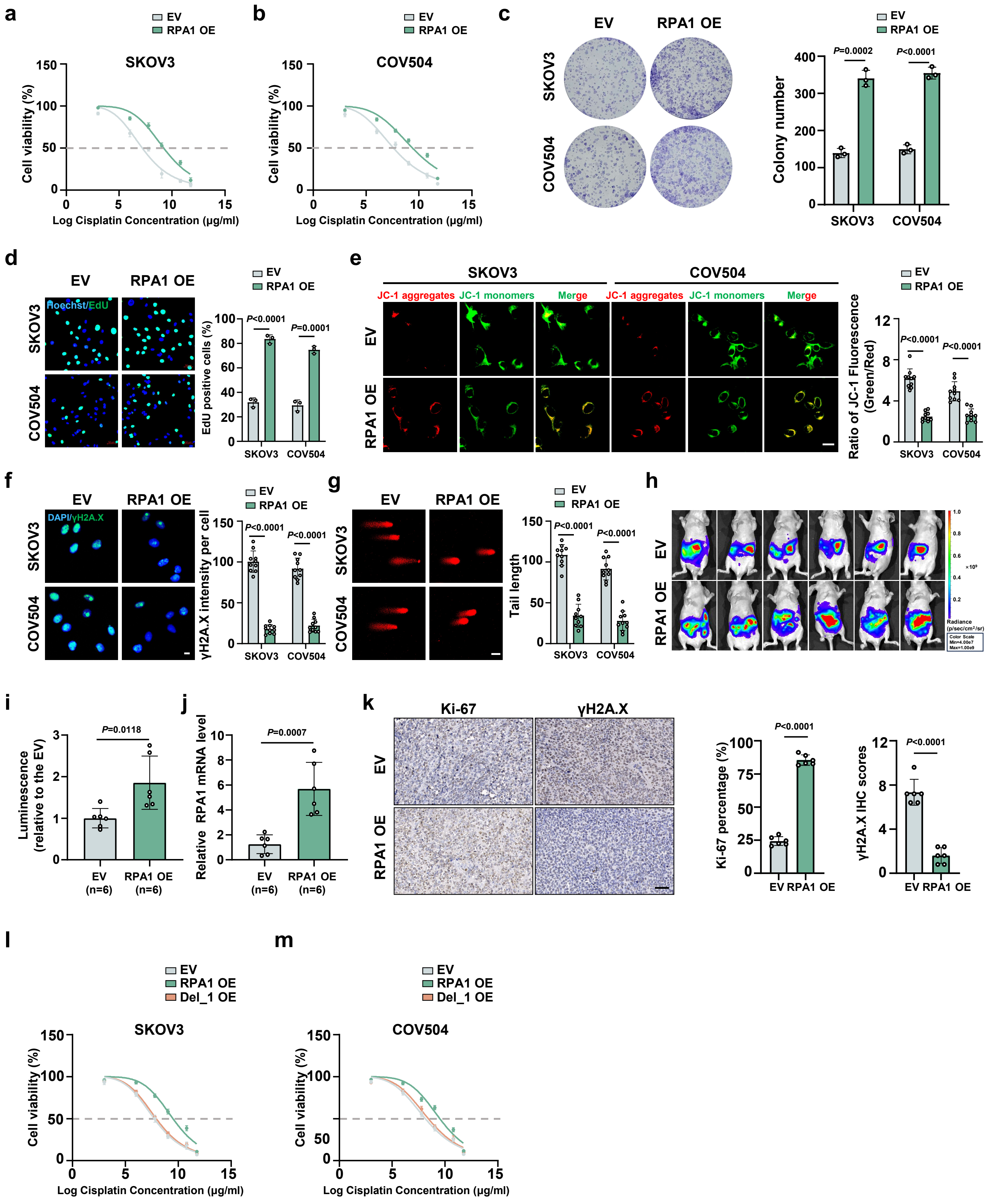

Supplementary figure 5

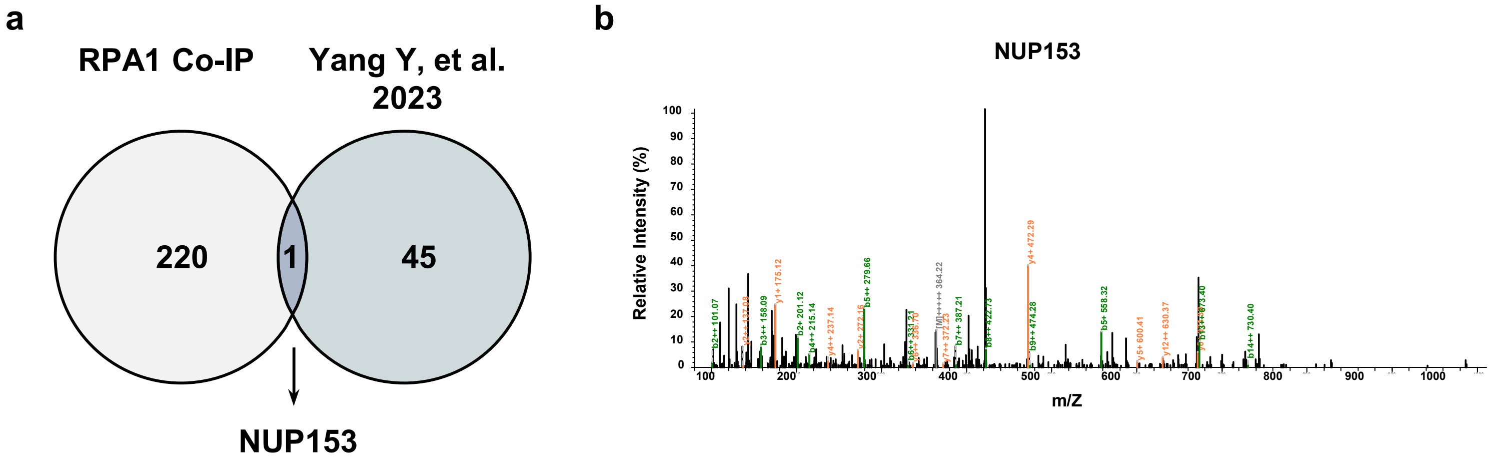

Supplementary figure 6

a

Protein Name	Unique Peptides	Sequence Coverage (%)
H2AFY	7	34.8
DDX18	6	14.3
DDX3	5	10.1

b

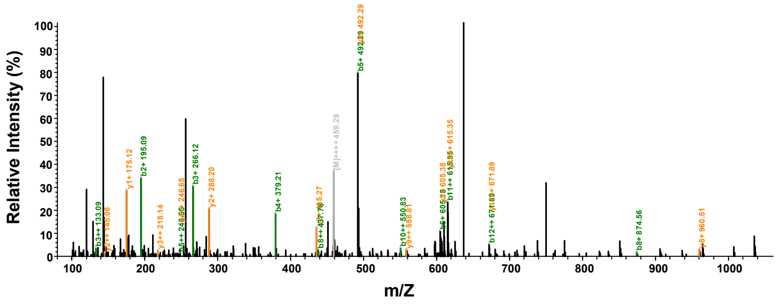

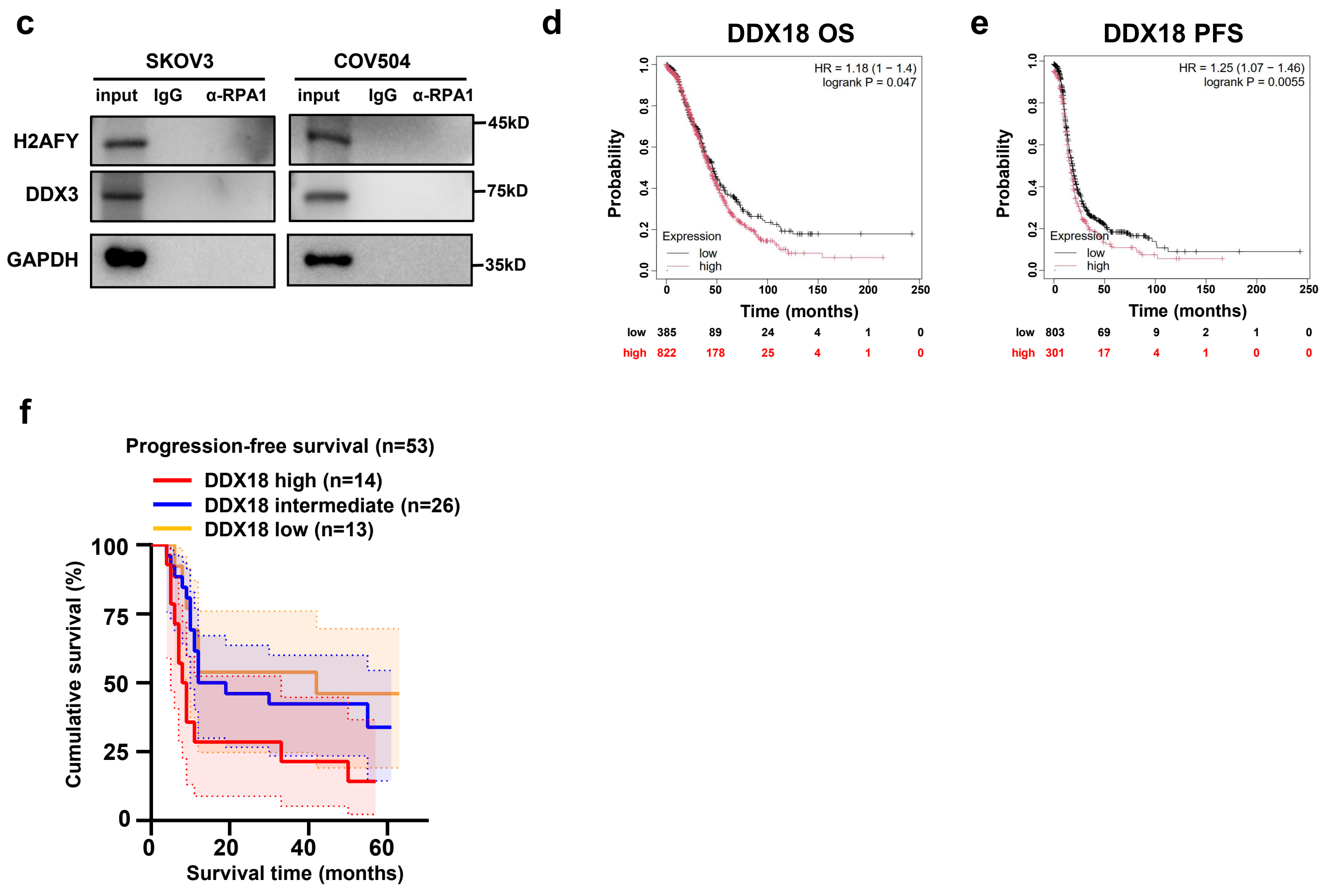

Supplementary figure 7

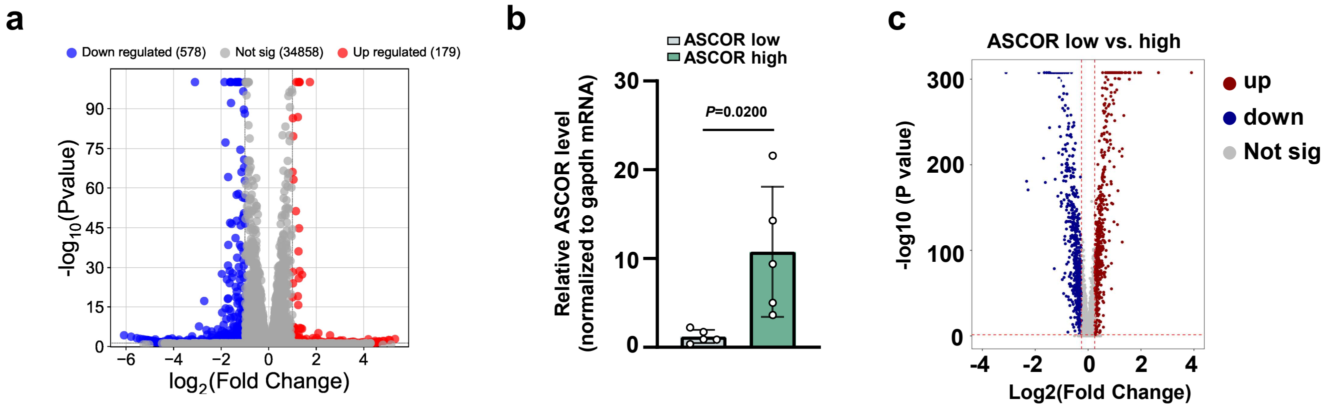

Supplementary figure 8

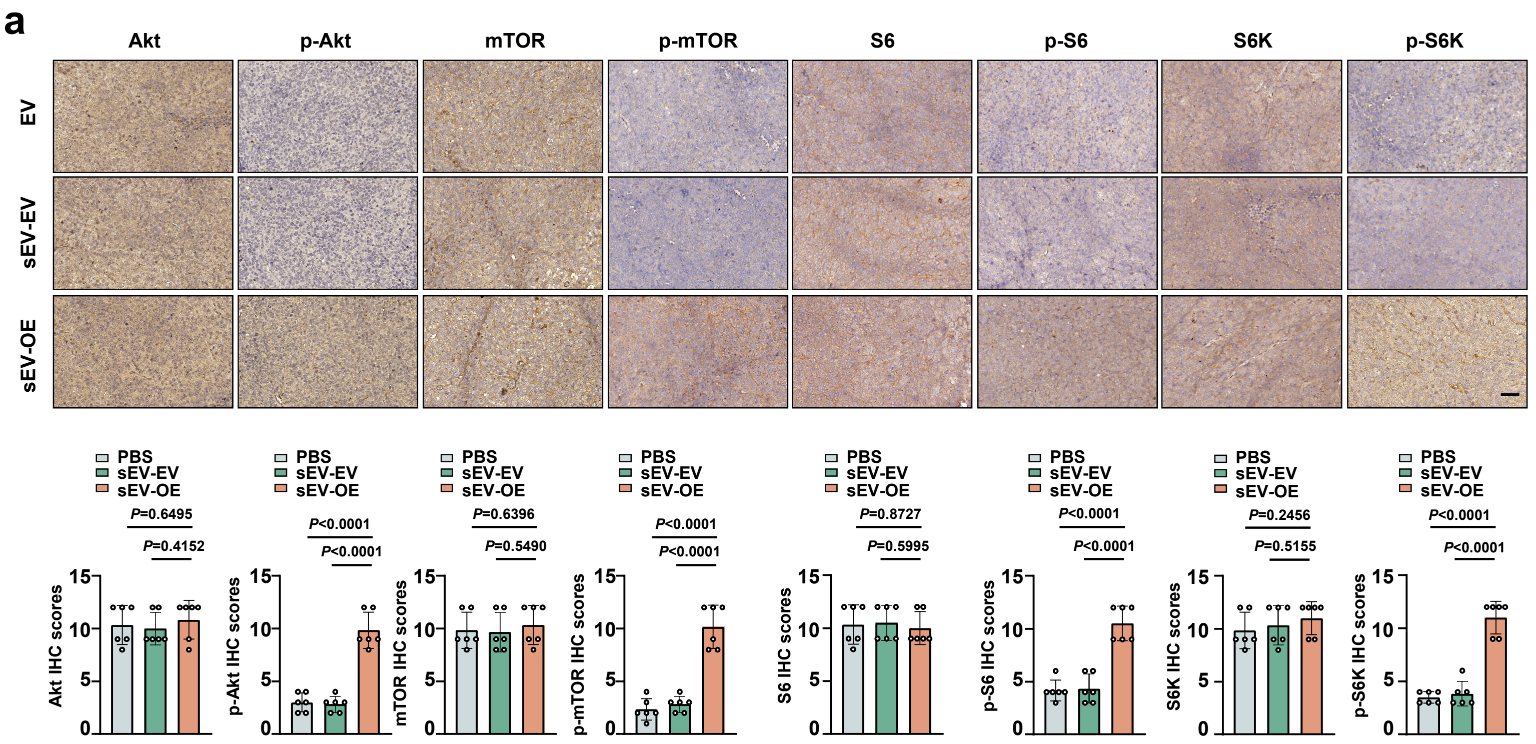

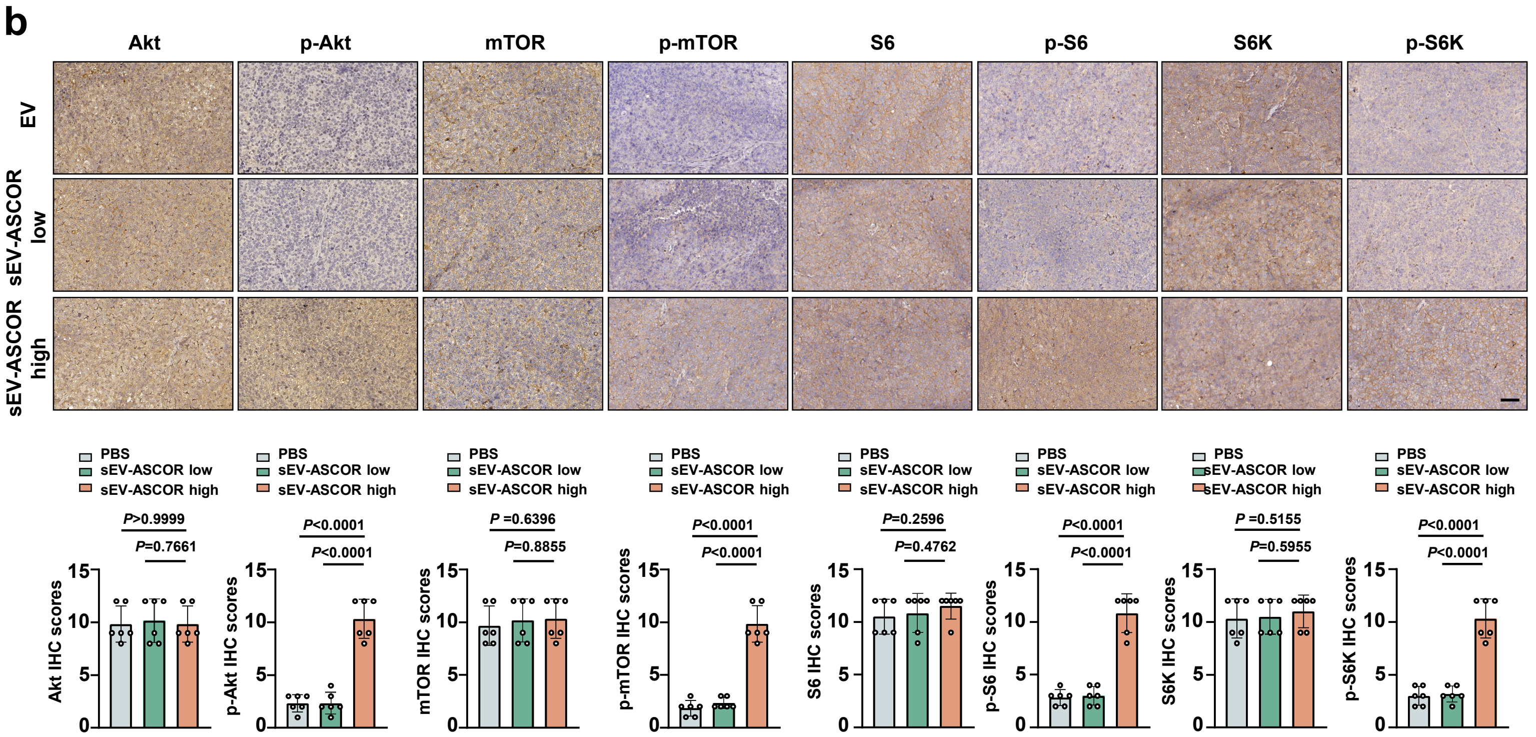

Supplementary figure 9

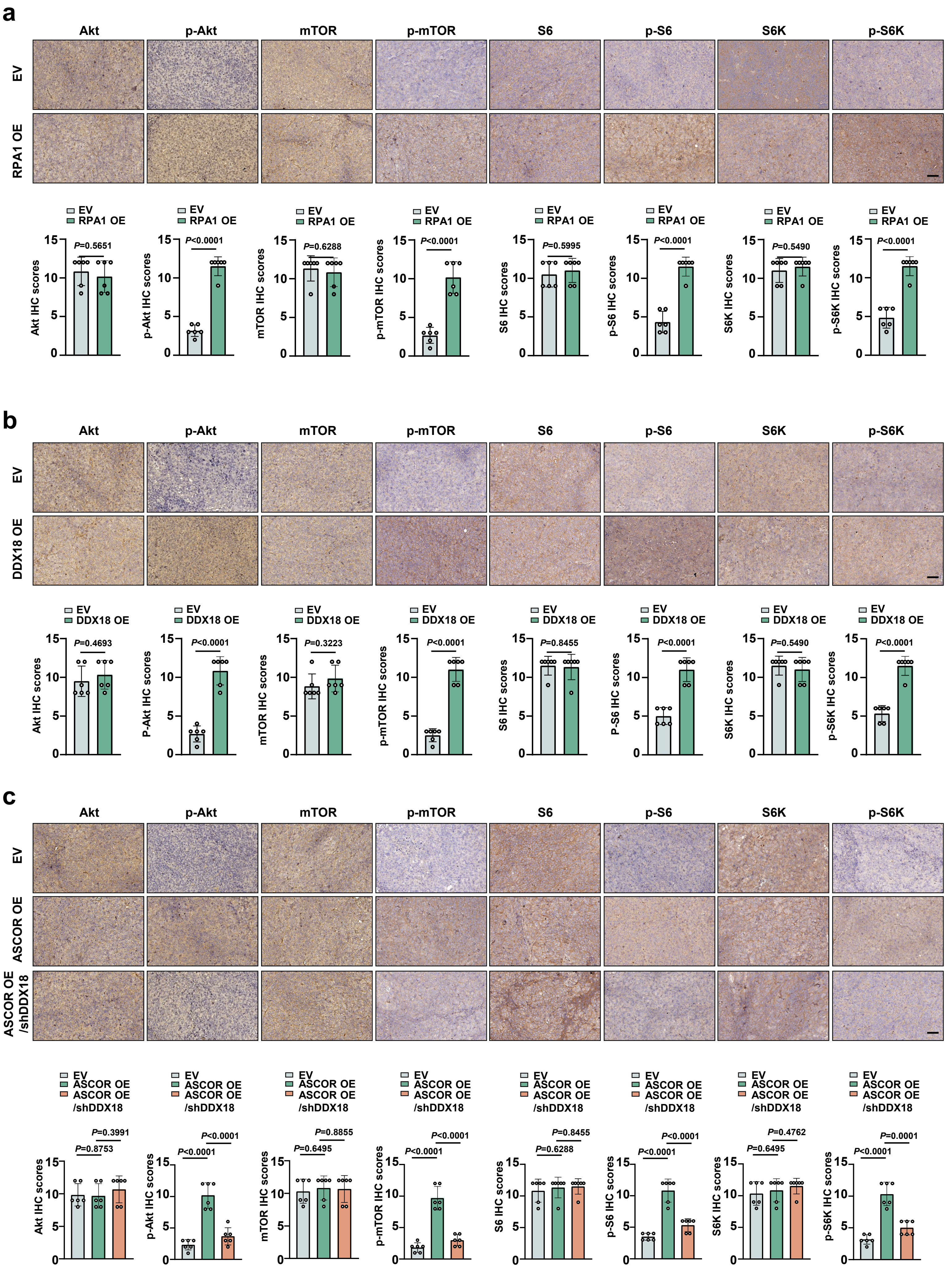

Supplement: Supplementary file 2 — Supporting File: advs74359‐sup‐0002‐Figure S1‐S9. [file ADVS-13-e18922-s001.pdf]
