# Supplementary material for: Ascites circRNA ASCOR Drives Platinum Resistance of High‐Grade Serous Ovarian Cancer by Facilitating RPA1 Nuclear Translocation
Source: Adv Sci (Weinh). 2026 Feb 12;13(23):e18922. doi: 10.1002/advs.202518922 (PMC13104110; doi:10.1002/advs.202518922)
Supplement: Supplementary file 3 — Supporting File: advs74359‐sup‐0003‐ Table S1‐S7.docx. [file ADVS-13-e18922-s002.docx]

**Supplemental Table 1. Clinical information of patients with RNA-seq in this study**

| Patient ID | Age (yrs) | Pathological diagnosis | FIGO stage | Ascite fluid volume(ml) |
| --- | --- | --- | --- | --- |
| S1 | 62 | HGSOC | ⅢC | 1100 |
| S2 | 47 | HGSOC | ⅢC | 1000 |
| S3 | 48 | HGSOC | ⅢC | 1200 |
| S4 | 59 | HGSOC | ⅢC | 2000 |
| S5 | 49 | HGSOC | ⅢC | 1200 |
| S6 | 62 | HGSOC | ⅢC | 1500 |
| S7 | 37 | HGSOC | ⅣA | 1800 |
| R1 | 49 | HGSOC | ⅢC | 1600 |
| R2 | 56 | HGSOC | ⅢC | 1400 |
| R3 | 49 | HGSOC | ⅢC | 1500 |
| R4 | 52 | HGSOC | ⅢB | 800 |
| R5 | 51 | HGSOC | ⅢC | 1000 |
| R6 | 63 | HGSOC | ⅢC | 1200 |
| R7 | 50 | HGSOC | ⅢC | 1300 |
| R8 | 52 | HGSOC | ⅡB | 500 |
| R9 | 52 | HGSOC | ⅢC | 1000 |
| R10 | 74 | HGSOC | ⅢB | 2000 |

**Supplemental Table 2. Patients’ clinical information for qRT-PCR validation** **of HGSOC ascites sEV, matched tumor tissues, omentum, and peritoneum tissues in this study**

| Patient ID | Age (yrs) | Pathological diagnosis | FIGO stage | Ascite fluid volume(ml) |
| --- | --- | --- | --- | --- |
| 1 | 68 | HGSOC | ⅢC | 1400 |
| 2 | 55 | HGSOC | ⅢC | 1500 |
| 3 | 68 | HGSOC | ⅡB | 1200 |
| 4 | 57 | HGSOC | ⅢC | 1400 |
| 5 | 60 | HGSOC | ⅢC | 900 |
| 6 | 57 | HGSOC | ⅢC | 1700 |
| 7 | 41 | HGSOC | ⅣB | 1300 |
| 8 | 58 | HGSOC | ⅢC | 1800 |
| 9 | 75 | HGSOC | ⅢA | 2100 |
| 10 | 47 | HGSOC | ⅢC | 1400 |
| 11 | 33 | HGSOC | ⅡB | 400 |

**Supplemental Table 3. Patients’ clinical information for qRT-PCR validation of HGSOC ascites sEV, matched tumor tissues in this study**

| Patient ID | Age (yrs) | Pathological diagnosis | FIGO stage | Ascite fluid volume(ml) | Relapse | Progression-free survival |
| --- | --- | --- | --- | --- | --- | --- |
| S1 | 44 | HGSOC | ⅢC | 1500 | Yes | 42 |
| S2 | 64 | HGSOC | Ⅳ | 1700 | No | 53 |
| S3 | 54 | HGSOC | ⅢC | 1200 | No | 50 |
| S4 | 51 | HGSOC | ⅢC | 1500 | No | 61 |
| S5 | 47 | HGSOC | ⅢC | 1000 | Yes | 33 |
| S6 | 37 | HGSOC | Ⅳ | 1900 | Yes | 50 |
| S7 | 43 | HGSOC | ⅢA | 1100 | No | 60 |
| S8 | 43 | HGSOC | ⅡB | 400 | No | 59 |
| S9 | 49 | HGSOC | ⅢA | 900 | No | 63 |
| S10 | 52 | HGSOC | ⅢC | 1200 | No | 58 |
| S11 | 53 | HGSOC | ⅡB | 1000 | No | 55 |
| S12 | 52 | HGSOC | ⅢA | 1500 | No | 55 |
| S13 | 48 | HGSOC | Ⅳ | 2200 | No | 55 |
| S14 | 52 | HGSOC | ⅢC | 1300 | No | 55 |
| S15 | 50 | HGSOC | ⅢC | 1500 | No | 53 |
| S16 | 55 | HGSOC | ⅣA | 2400 | No | 52 |
| S17 | 58 | HGSOC | Ⅳ | 1800 | No | 52 |
| S18 | 48 | HGSOC | ⅢC | 1200 | No | 51 |
| S19 | 62 | HGSOC | ⅣB | 2000 | Yes | 30 |
| S20 | 55 | HGSOC | ⅢC | 1400 | Yes | 19 |
| S21 | 48 | HGSOC | ⅣB | 2100 | No | 52 |
| S22 | 51 | HGSOC | ⅢA | 1000 | No | 58 |
| S23 | 46 | HGSOC | ⅢC | 2100 | No | 57 |
| S24 | 53 | HGSOC | ⅡB | 1600 | No | 59 |
| R1 | 52 | HGSOC | ⅢC | 1600 | Yes | 9 |
| R2 | 58 | HGSOC | ⅢC | 1400 | Yes | 8 |
| R3 | 59 | HGSOC | ⅢC | 1500 | Yes | 12 |
| R4 | 53 | HGSOC | ⅢC | 1800 | Yes | 11 |
| R5 | 49 | HGSOC | ⅢC | 1700 | Yes | 9 |
| R6 | 55 | HGSOC | ⅢC | 800 | Yes | 6 |
| R7 | 61 | HGSOC | ⅢC | 600 | Yes | 4 |
| R8 | 54 | HGSOC | ⅢC | 1400 | Yes | 7 |
| R9 | 63 | HGSOC | ⅢC | 900 | Yes | 10 |
| R10 | 57 | HGSOC | ⅢB | 2200 | Yes | 12 |
| R11 | 55 | HGSOC | ⅢC | 1400 | Yes | 9 |
| R12 | 52 | HGSOC | ⅢA | 1100 | Yes | 7 |
| R13 | 41 | HGSOC | ⅢA | 1800 | Yes | 8 |
| R14 | 51 | HGSOC | ⅢA | 1000 | Yes | 12 |
| R15 | 49 | HGSOC | ⅢC | 1600 | Yes | 5 |
| R16 | 48 | HGSOC | ⅢA | 1700 | Yes | 5 |
| R17 | 74 | HGSOC | ⅢB | 1400 | Yes | 4 |
| R18 | 59 | HGSOC | ⅢC | 1300 | Yes | 10 |
| R19 | 55 | HGSOC | ⅢC | 1800 | Yes | 6 |
| R20 | 54 | HGSOC | ⅢC | 1700 | Yes | 8 |
| R21 | 49 | HGSOC | ⅢC | 2400 | Yes | 9 |
| R22 | 66 | HGSOC | ⅢB | 1500 | Yes | 12 |
| R23 | 58 | HGSOC | ⅢB | 1100 | Yes | 11 |
| R24 | 52 | HGSOC | ⅡB | 500 | Yes | 6 |
| R25 | 50 | HGSOC | ⅢC | 2100 | Yes | 10 |
| R26 | 67 | HGSOC | ⅢC | 2400 | Yes | 10 |
| R27 | 65 | HGSOC | ⅢC | 1600 | Yes | 11 |
| R28 | 69 | HGSOC | ⅢB | 1800 | Yes | 12 |
| R29 | 62 | HGSOC | ⅢC | 2300 | Yes | 5 |

**Supplemental Table 4. Clinical information for snRNA-seq validation in this study**

| Patient ID | Age (yrs) | Pathological diagnosis | FIGO stage | Relapse | Progression-free survival |
| --- | --- | --- | --- | --- | --- |
| ASCOR high1 | 51 | HGSOC | ⅢC | Yes | 8 |
| ASCOR high2 | 63 | HGSOC | ⅢC | Yes | 18 |
| ASCOR high3 | 56 | HGSOC | ⅢC | Yes | 21 |
| ASCOR high4 | 46 | HGSOC | ⅣA | Yes | 14 |
| ASCOR high5 | 71 | HGSOC | ⅣA | Yes | 9 |
| ASCOR low1 | 63 | HGSOC | ⅢC | No | 29 |
| ASCOR low2 | 52 | HGSOC | ⅢC | No | 31 |
| ASCOR low3 | 61 | HGSOC | ⅣA | No | 35 |
| ASCOR low4 | 63 | HGSOC | ⅢB | No | 27 |
| ASCOR low5 | 70 | HGSOC | ⅡB | No | 31 |

**Supplemental Table 5. Antibodies used in this study**

| Antibody | Manufacturer | product code | RRIDs |
| --- | --- | --- | --- |
| Anti-Alix | Proteintech | 12422-1-AP | AB_2162467 |
| Anti-HSP70 | Proteintech | 10995-1-AP | AB_2264230 |
| Anti-CD9 | Proteintech | 20597-1-AP | AB_2878706 |
| Anti-CD81 | Proteintech | 27855-1-AP | AB_2880995 |
| Anti-TSG101 | abcam | ab125011 | AB_10974262 |
| Anti-Calnexin | Proteintech | 10427-2-AP | AB_2069033 |
| Anti-GM130 | Proteintech | 11308-1-AP | AB_2115327 |
| Anti-RPA1 | abcam | ab79398 | AB_1603759 |
| Anti-DDX18 | Proteintech | 28502-1-AP | AB_2881158 |
| Anti-H2AFY | Proteintech | 26875-1-AP | AB_2918113 |
| Anti-DDX3 | Proteintech | 11115-1-AP | AB_10896499 |
| Anti-Histone H3 | Proteintech | 17168-1-AP | AB_2716755 |
| Anti-NUP153 | Proteintech | 14189-1-AP | AB_2154463 |
| Anti-Akt | Proteintech | 10176-2-AP | AB_2224574 |
| Anti-PI3 Kinase p85 Alpha | Proteintech | 60225-1-Ig | AB_11042594 |
| Anti-Phospho-Akt (Ser473) | Proteintech | 66444-1-Ig | AB_2782958 |
| Anti-mTOR | Proteintech | 66888-1-Ig | AB_2882219 |
| Anti-Phospho-mTOR (Ser2448) | Proteintech | 80596-1-RR | AB_2918905 |
| Anti-S6 | Proteintech | 66886-1-Ig | AB_2882218 |
| Anti-Phospho-S6(Ser235/236) | CST | 2211S | AB_331679 |
| Anti-S6K | Proteintech | 14485-1-AP | AB_2269787 |
| Anti-Phospho-S6K(Thr389/412) | Proteintech | 28735-1-AP | AB_2918197 |
| Anti- HNRNPA2B1 | Proteintech | 14813-1-AP | AB_2279638 |
| Anti-βTubulin | Proteintech | 10094-1-AP | AB_2210695 |
| Anti-GAPDH | Proteintech | 60004-1-Ig | AB_2107436 |
| Anti-FLAG | Proteintech | 20543-1-AP | AB_11232216 |
| Anti-S9.6 | Karafast | ENH001 | AB_2687463 |
| Anti-Phospho-Histone H2A.X (Ser139) | CST | 80312S | AB_2799949 |

**Supplemental Table 6. Sequences of primers and probes used in this study**

| Application | Name | Sequences | |
| --- | --- | --- | --- |
| qRT-PCR | ASCOR | 5’-3’ | TTGTCAAAAGGAAGCCCTTG |
|  |  | 3’-5’ | TAAACTCTGGCCCCTTCCTC |
| qRT-PCR | RPA1 | 5’-3’ | ACATCCGTCCCATTTCTACAGG |
|  |  | 3’-5’ | CTCCCTCGACCAGGGTGTT |
| qRT-PCR | DDX18 | 5’-3’ | CTTGGCCATTCATGGAAAGCA |
|  |  | 3’-5’ | GGCTGTTCTACCCACACGAT |
| qRT-PCR | GAPDH | 5’-3’ | AACGGATTTGGTCGTATTGGG |
|  |  | 3’-5’ | CCTGGAAGATGGTGATGGGAT |
| qRT-PCR | U1 | 5’-3’ | GATACCATGATCACGAAGGTG |
|  |  | 3’-5’ | CTACCACAAATTATGCAGTCG |
| RNA pulldown | ASCOR | 5’-3’ | TCCTGTTTGGTTTGGTTCCAATTTATGCTC |
| RNA pulldown | Scramble | 5’-3’ | TTCTCCGAACGTGTCACGTTCGAACGTGTC |
| RNA FISH | ASCOR | 5’-3’ | TTTGGTTTGGTTCCAATTTATGCTC |

**Supplemental Table 7. Sequences of siRNAs in this study**

| **siRNA** | **Sequence** | |
| --- | --- | --- |
| NUP153  siRNA | Sense (5’-3’) | GGCAGUCCCAUGUUUAAAUTT |
|  | Anti-sense (3’-5’) | AUUUAAACAUGGGACUGCCTT |
| HNRNPA2B1  siRNA | Sense (5’-3’) | GGCUUUGUCUAGACAAGAATT |
|  | Anti-sense (3’-5’) | UUCUUGUCUAGACAAAGCCTT |
